# Intracellular accumulation of hydrophobic 1,3-PDN inhibits methanogenesis in rumen methanogens

**DOI:** 10.1093/ismejo/wrag200

**Published:** 2026-07-28

**Authors:** Bingzi Ouyang, Yurong Cao, Qiushuang Li, Shizhe Zhang, Xiang Zhou, Zhiwu Wu, Xinyi Li, Cancan Wang, Rong Wang, Bo Fu, Xiaojia Liu, Lixiang Yin, Zhiliang Tan, Kui Zhu, Le Luo Guan, Xiumin Zhang, Jie Li, Min Wang

**Affiliations:** State Key Laboratory of Forage Breeding-by-Design and Utilization, National Engineering Laboratory for Pollution Control and Waste Utilization in Livestock and Poultry Production, Institute of Subtropical Agriculture, Chinese Academy of Sciences, Changsha, Hunan 410125, China; College of Advanced Agricultural Sciences, University of Chinese Academy of Sciences, Beijing 100049, China; State Key Laboratory of Forage Breeding-by-Design and Utilization, National Engineering Laboratory for Pollution Control and Waste Utilization in Livestock and Poultry Production, Institute of Subtropical Agriculture, Chinese Academy of Sciences, Changsha, Hunan 410125, China; College of Advanced Agricultural Sciences, University of Chinese Academy of Sciences, Beijing 100049, China; State Key Laboratory of Forage Breeding-by-Design and Utilization, National Engineering Laboratory for Pollution Control and Waste Utilization in Livestock and Poultry Production, Institute of Subtropical Agriculture, Chinese Academy of Sciences, Changsha, Hunan 410125, China; State Key Laboratory of Forage Breeding-by-Design and Utilization, National Engineering Laboratory for Pollution Control and Waste Utilization in Livestock and Poultry Production, Institute of Subtropical Agriculture, Chinese Academy of Sciences, Changsha, Hunan 410125, China; College of Advanced Agricultural Sciences, University of Chinese Academy of Sciences, Beijing 100049, China; State Key Laboratory of Forage Breeding-by-Design and Utilization, National Engineering Laboratory for Pollution Control and Waste Utilization in Livestock and Poultry Production, Institute of Subtropical Agriculture, Chinese Academy of Sciences, Changsha, Hunan 410125, China; College of Advanced Agricultural Sciences, University of Chinese Academy of Sciences, Beijing 100049, China; State Key Laboratory of Forage Breeding-by-Design and Utilization, National Engineering Laboratory for Pollution Control and Waste Utilization in Livestock and Poultry Production, Institute of Subtropical Agriculture, Chinese Academy of Sciences, Changsha, Hunan 410125, China; College of Advanced Agricultural Sciences, University of Chinese Academy of Sciences, Beijing 100049, China; State Key Laboratory of Forage Breeding-by-Design and Utilization, National Engineering Laboratory for Pollution Control and Waste Utilization in Livestock and Poultry Production, Institute of Subtropical Agriculture, Chinese Academy of Sciences, Changsha, Hunan 410125, China; College of Advanced Agricultural Sciences, University of Chinese Academy of Sciences, Beijing 100049, China; State Key Laboratory of Forage Breeding-by-Design and Utilization, National Engineering Laboratory for Pollution Control and Waste Utilization in Livestock and Poultry Production, Institute of Subtropical Agriculture, Chinese Academy of Sciences, Changsha, Hunan 410125, China; College of Advanced Agricultural Sciences, University of Chinese Academy of Sciences, Beijing 100049, China; State Key Laboratory of Forage Breeding-by-Design and Utilization, National Engineering Laboratory for Pollution Control and Waste Utilization in Livestock and Poultry Production, Institute of Subtropical Agriculture, Chinese Academy of Sciences, Changsha, Hunan 410125, China; College of Advanced Agricultural Sciences, University of Chinese Academy of Sciences, Beijing 100049, China; Jiangsu Key Laboratory for Recognition and Remediation of Emerging Pollutants in Taihu Basin, School of Environmental Science and Engineering, Wuxi University, Wuxi, Jiangsu 214105, China; State Key Laboratory of Veterinary Public Health and Safety, College of Veterinary Medicine, China Agricultural University, Beijing 100193, China; State Key Laboratory of Veterinary Public Health and Safety, College of Veterinary Medicine, China Agricultural University, Beijing 100193, China; State Key Laboratory of Forage Breeding-by-Design and Utilization, National Engineering Laboratory for Pollution Control and Waste Utilization in Livestock and Poultry Production, Institute of Subtropical Agriculture, Chinese Academy of Sciences, Changsha, Hunan 410125, China; College of Advanced Agricultural Sciences, University of Chinese Academy of Sciences, Beijing 100049, China; State Key Laboratory of Veterinary Public Health and Safety, College of Veterinary Medicine, China Agricultural University, Beijing 100193, China; Faculty of Land and Food Systems, The University of British Columbia, Vancouver, BC V6T 1Z4, Canada; State Key Laboratory of Forage Breeding-by-Design and Utilization, National Engineering Laboratory for Pollution Control and Waste Utilization in Livestock and Poultry Production, Institute of Subtropical Agriculture, Chinese Academy of Sciences, Changsha, Hunan 410125, China; College of Advanced Agricultural Sciences, University of Chinese Academy of Sciences, Beijing 100049, China; State Key Laboratory of Microbial Diversity and Innovative Utilization, Institute of Microbiology, Chinese Academy of Sciences, Beijing 100101, China; State Key Laboratory of Forage Breeding-by-Design and Utilization, National Engineering Laboratory for Pollution Control and Waste Utilization in Livestock and Poultry Production, Institute of Subtropical Agriculture, Chinese Academy of Sciences, Changsha, Hunan 410125, China; College of Advanced Agricultural Sciences, University of Chinese Academy of Sciences, Beijing 100049, China

**Keywords:** methane production, methanogenesis inhibitor, methyl-coenzyme M reductase, hydrogen metabolism

## Abstract

Methane (CH_4_) from ruminants is a major source of agricultural greenhouse gas and represents a loss of dietary energy. 3-Nitrooxypropanol (3-NOP) is a known methanogenesis inhibitor, but its hydrophilic nature may limit the cellular accessibility to methanogens. Here, we systematically evaluated 1,3-propanediol dinitrate (1,3-PDN), a more hydrophobic derivative of 3-NOP, for its antimethanogenic potential and underlying mode of action using ruminal fermentation, pure-culture assays, multi-omics analyses, and molecular docking. Intracellular accumulation assays indicated greater cellular accumulation of 1,3-PDN than 3-NOP in rumen-derived methanogen *Methanobrevibacter olleyae*. Ruminal fermentation assays showed that 1,3-PDN reduced CH_4_ production by ~55%, and altered hydrogen (H_2_) metabolism, leading to 11-fold increase in H_2_ accumulation. Metatranscriptomic profiling revealed that 1,3-PDN significantly altered the active archaeal community, with a notable reduction in *Methanobrevibacter_A* and suppression of hydrogenotrophic methanogenesis. Molecular docking suggested that 1,3-PDN may bind to the conserved active site of methyl-coenzyme M reductase (MCR), potentially contributing to MCR-associated inhibition. Proteomic analysis further indicated that 1,3-PDN supplementation downregulated key MCR subunits and simultaneously affected other redox-sensitive methanogenesis-related processes, including tetrahydromethanopterin S-methyltransferase subunits and proteins involved in the biosynthesis of cofactors F_430_ and cobalamin. Nitrogen-equivalent assay suggested partial contribution of nitrite to the methanogenesis inhibition and oxidative effects induced by 1,3-PDN. Together, these findings identify 1,3-PDN as an effective inhibitor of ruminal methanogenesis with enhanced cellular enrichment, providing mechanistic insights for the rational development of new CH_4_ inhibitors.

## Introduction

Methane (CH_4_), a potent greenhouse gas (GHG), accounts for ~21% of global radiative forcing, with atmospheric concentrations rising by over 32.4% since 1990 [1, 2]. Enteric fermentation in ruminants accounts for ~65%–70% of agricultural CH_4_ emissions [3, 4] and represents a loss of dietary energy to the host animal, ranging from 2% to 12% of gross energy intake [5, 6]. Consequently, mitigating CH_4_ emissions from ruminants is critical for both environmental sustainability and livestock feed efficiency.

Methanogenesis in the rumen is primarily driven by methanogenic archaea, which convert substrates such as hydrogen (H_2_), carbon dioxide (CO_2_), methanol, methylamines, and acetate into CH_4_ through a series of complex metabolic pathways [7]. Across all these methanogenic pathways, methyl-coenzyme M reductase (MCR), a cytoplasmic enzyme, catalyzes the terminal step of CH_4_ biosynthesis and is a key enzyme conserved across all known methanogenic metabolisms [8]. Targeting MCR activity with small molecules is therefore crucial for the development of inhibitors [9]. To be effective, these inhibitors must first traverse the cell membrane, as their action relies on reaching the active site of MCR. 3-Nitrooxypropanol (3-NOP), a well-studied MCR inhibitor, is proposed to inactivate MCR by oxidizing the nickel (Ni) ion in the F_430_ cofactor, thereby blocking catalysis [10], and can reduce CH_4_ emissions by >30% without compromising animal health and productivity [11, 12].

In rational inhibitor design, the hydrophobicity of small-molecule compounds is a key determinant of transmembrane permeability. Generally, moderate hydrophobicity facilitates diffusion across biological membranes and promotes accumulation at target sites, thereby enabling effective pharmacological activity [13]. Unlike bacterial membranes, archaeal cellular membranes are composed of ether-linked lipids, a unique structural feature that confers high hydrophobicity and low permeability [14, 15] As a hydrophilic compound, 3-NOP is rapidly diluted and washed out of the rumen [11], which may limit its membrane penetration and sustained inhibitory effect on MCR. Its hydroxyl (–OH) group is the major contributor to this hydrophilicity. Replacing the –OH with nitrate ester (–ONO_2_) generates 1,3-propanediol dinitrate (1,3-PDN) (Supplementary Fig. S1A), a more hydrophobic structural analog, that may substantially alter the compound’s transmembrane permeability. We hypothesized that this modification could favor cellular access to methanogenic cells. However, such a structural modification may also change its interaction with MCR, which warrants further mechanistic evaluation.

Here, we evaluated the antimethanogenic potential of 1,3-PDN and investigated its possible mode of action using integrated experimental and computational approaches. We first compared the intracellular accumulation of 1,3-PDN and 3-NOP in the rumen-derived methanogen *Methanobrevibacter olleyae*, and then assessed the inhibitory effects of 1,3-PDN through ruminal fermentation assays, multi-omics analyses, and molecular docking. We demonstrate that 1,3-PDN achieves greater intracellular accumulation than 3-NOP in *M. olleyae* and substantially suppresses ruminal hydrogenotrophic methanogenesis. Our data support a working model in which 1,3-PDN-mediated inhibition is associated with potential MCR-related effects and broader perturbation of redox-sensitive methanogenesis-related processes, including the MTR complex, cofactor F₄₃₀ biosynthesis, and cobalamin biosynthesis pathways. Together, these findings identify 1,3-PDN as an effective inhibitor of CH_4_ production with enhanced cellular enrichment, offering novel insights into the molecular design of optimized CH_4_ inhibitors and establishing a theoretical basis for future investigations into their potential on-farm application.

## Materials and methods

### Chemical synthesis and reagents

1,3-Propanediol dinitrate (1,3-PDN) was synthesized from 1,3-dibromopropane and silver nitrate, following the procedures as previously described [16]. The final product purity of 1,3-PDN was determined using the method described by Ünsal *et al*. [17] and was ~94.5% (Supplementary Fig. S2), with polynitrate esters identified as the principal impurities. 1,3-dibromopropane, silver nitrate, sodium nitrite, and sodium nitrate were obtained from Sinopharm Chemical Reagent Co., Ltd. 3-NOP was purchased from TargetMol Chemicals Inc.

### Isolation of a hydrogenotrophic methanogen *M. olleyae* from rumen fluid

A hydrogenotrophic methanogenic strain was isolated from the rumen fluid of a ruminal-cannulated Hu sheep under strict anoxic conditions using a gas mixture of H_2_/CO_2_ (80:20, v/v). Isolation and purification were performed using a modified roll-tube technique based on the Hungate method [18]. Detailed procedures for enrichment, serial transfer, and colony purification are provided in the Supplementary Materials and Methods. After the final round, the purified methanogenic isolate was identified via 16S rRNA genes Sanger sequencing using primers Met86F (5′-GCTCAGTAACACGTGG-3′) and Met1340R (5′-CGGTGTGTGCAAGGAG-3′) [19]. The resulting sequence was compared to reference sequences in the NCBI database using BLAST [20], indicating the purified methanogenic isolate showed 99.75% sequence identity with *M. olleyae* and belonged to the highly abundant RO clade of archaeal community in rumen microbial ecosystem (Supplementary File S1, Supplementary Fig. S3). Fourteen 16S rRNA sequences of methanogens from NCBI database closely related to the methanogenic isolate obtained in this study were selected as reference sequences and used for constructing the phylogenetic tree using the neighbor-joining algorithm within MEGA [21] (version 11) under default parameters.

### Intracellular accumulation assay of inhibitors in *M. olleyae* cell

**Figure 1:**
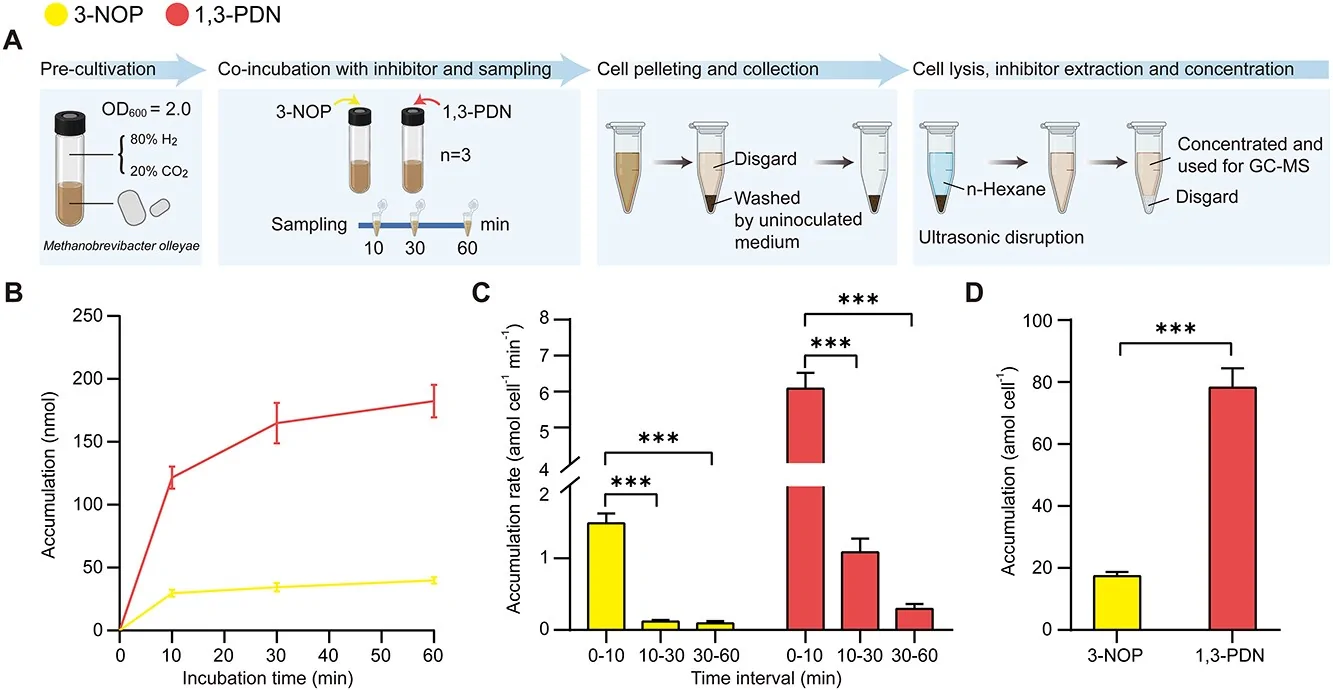
Intracellular accumulation of 1,3-PDN or 3-NOP in the *M. olleyae* from the rumen of Hu sheep. (A) Experimental workflow for quantifying intracellular accumulation of 3-NOP or 1,3-PDN in *M. olleyae*; (B) time-course intracellular accumulation abundance of each inhibitor in *M. olleyae* following exposure; (C) apparent accumulation rates calculated over the indicated time intervals in *M. olleyae*. (D) Mean intracellular accumulation level of each inhibitor in *M. olleyae* across the three sampling time points. Data with error bars are expressed as mean ± standard error. ^*^P < .05, ^**^P < .01, ^***^P < .001, *n* = 3; 3-NOP, 3-nitrooxypropanol; 1,3-PDN, 1,3-propanediol dinitrate; *M. olleyae, Methanobrevibacter olleyae*. The centrifuge tube model was created using BioRender.

The hydrogenotrophic methanogen strain, *M. olleyae*, was used to quantify the intracellular accumulation of 1,3-PDN or 3-NOP (Fig. 1A, Experiment 1). *Methanobrevibacter olleyae* was cultivated in 300 ml of pre-reduced medium, under a gas phase of H_2_/CO_2_ (80:20, v/v) at 39°C. After 48 h of incubation, cultures reached the exponential phase and were centrifugated (4000 × *g*, 10 min, room temperature) to concentrate the cells 10-fold, resulting in a methanogenic suspension with OD_600_ of 2.00.

For inhibitor exposure, 0.1 ml of a 1 M 1,3-PDN or 3-NOP solution was added to 3.9 ml of the concentrated *M. olleyae* culture to obtain a final concentration of 25 mM. Incubations were conducted at 39°C under H_2_/CO_2_ (80:20, v/v). Aliquots (1 ml) were collected at 10, 30, and 60 min after the start of incubation. Cells were harvested by centrifugation (4000 × *g* for 10 min) and washed three times with sterile, uninoculated culture medium of the same formulation to remove extracellular compounds. Subsequently, cell pellets were lysed by ultrasonication on ice until complete disruption. Intracellular inhibitors were extracted from lysates using hexane. Following centrifugation (10 000 × *g* for 5 min at 4°C), the supernatant was collected and concentrated to a final volume of 150 μl by nitrogen evaporator for subsequent quantification.

Concentrations of 1,3-PDN or 3-NOP in the supernatant were determined by gas chromatography–mass spectrometry (GC-MS, Shimadzu GCMS-QP2020 NX) according to Yang *et al*. [16]. Specifically, the ion chromatograms for 3-NOP and 1,3-PDN were extracted in the SCAN mode, with retention times being 16.78 and 28.55 min, respectively (Supplementary Fig. S4A). The 3-NOP was elucidated based on the product ions of *m/z* 121 > 31, *m/z* 121 > 46, and *m/z* 121 > 76 (Supplementary Fig. S4B), whereas 1,3-PDN was elucidated based on the product ions of *m/z* 166 > 46 and *m/z* 166 > 76 (Supplementary Fig. S4C). Both 3-NOP and 1,3-PDN were analyzed using the same heating program (Supplementary Fig. S5).

### Methanogenesis inhibition through ruminal bath culture

All sheep involved in this study were reviewed and approved by the Animal Care Committee (approval number ISA-2025-0041), Institute of Subtropical Agriculture, the Chinese Academy of Sciences, Changsha, China.

**Figure 2:**
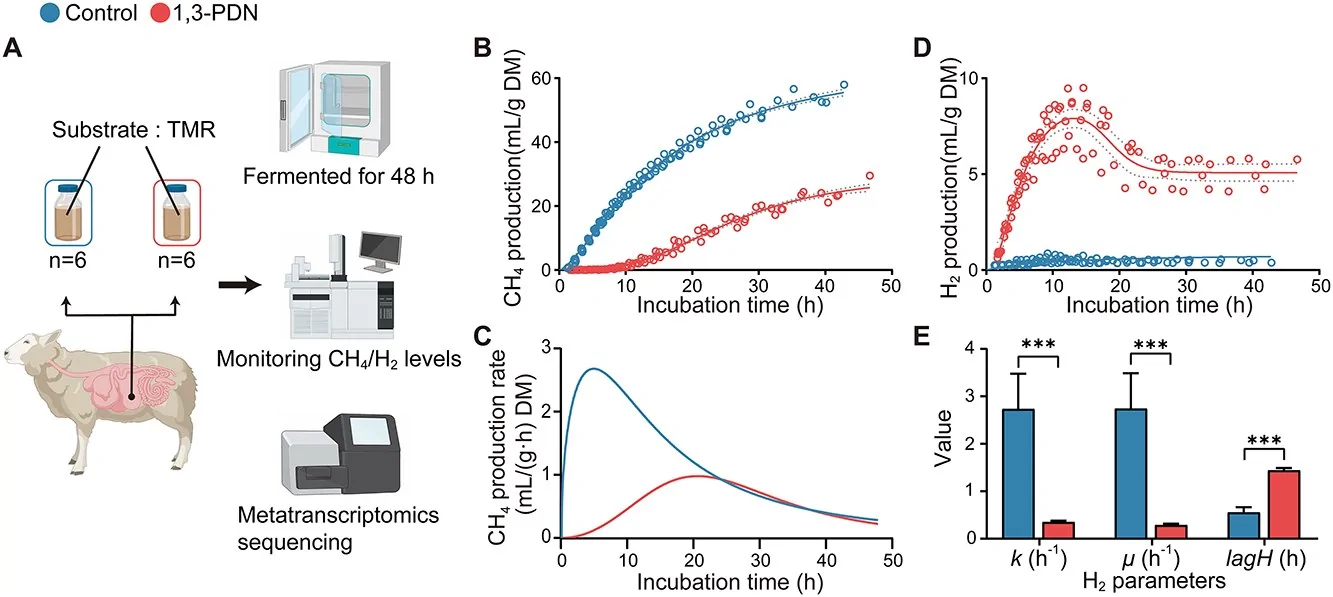
Effect of 1,3-PDN on the kinetics of methane (CH_4_) and hydrogen (H_2_) production through 48 h of ruminal batch culture. (A) Experimental design. Under anaerobic conditions, total mixed ration (TMR) substrates were incubated with sheep rumen inoculum to obtain the kinetics of CH_4_ and H_2_ production for 48 h, and microbial samples were collected for metatranscriptomic analysis; (B) kinetics of CH_4_ accumulation (ml/g DM); (C) rate of CH_4_ production (ml/g/h); (D) kinetics of H_2_ production (ml/g DM). (E) Parameters for the kinetics of H_2_ accumulation; *k*, fractional rate of H_2_ formation (h^−1^); *μ*, fractional rate of H_2_ re-solution (h^−1^); *lagH*, the lag time of H_2_ accumulation (h). Hollow circles represent gas measurements at different time points; the solid line shows the nonlinear fit, whereas the area enclosed by dashed lines indicates a 95% confidence interval; ^***^P < .001, *n* = 6; 1,3-PDN, 1,3-propanediol dinitrate; DM, dry matter; data with error bars are expressed as mean ± standard error. The schematic diagram was created using BioRender.

Ruminal batch culture was employed to quantify the inhibitive effect of 1,3-PDN on rumen methanogenesis (Fig. 2A, Experiment 2). Rumen contents were collected from two of three healthy adult ruminal-cannulated sheep (Hu, local breed, average BW 25 ± 2.0 kg) before morning feeding. The sheep were fed a total mixed ration (TMR), which included forage content (30%) and concentrate (70%) (dry matter basis, Supplementary Table S1). The rumen inoculum was obtained by filtering the rumen contents through four layers of sterile cheesecloth into a prewarmed insulated container. The filtered rumen fluid was subsequently mixed with artificial saliva [22] at a ratio of 1:4 (rumen fluid: saliva) to generate buffered rumen fluid.

Approximately 0.6 g of TMR was accurately weighed into 140 ml serum bottles, after which 60 ml of buffered rumen fluid was added under continuous flushing with CO_2_. A 0.12 mg (i.e. 0.722 μmol) of 1,3-PDN was supplemented to each bottle to obtain a concentration of 200 mg/kg (≙12 μM in the rumen fluid incubation system). Bottles were immediately sealed with butyl rubber stoppers, and incubations were conducted at 39.5°C using an automated *in vitro* batch incubation system with venting pressure maintained at 10.0 kPa, as described previously [23]. The vented gases were directed into a gas chromatograph (Agilent 7890A, Agilent Inc., Palo Alto, CA, USA) to quantify CH_4_ and H_2_ gas concentrations. The ruminal batch culture was ended after 48 h of incubation. Cumulative CH_4_ and H_2_ gas volumes were calculated based on the equations as previously outlined [24]. For fermentation-end products analysis, 2 ml of culture was centrifuged at 12 000 × *g* for 10 min at 4°C, and the supernatant was harvested for volatile fatty acid (VFA) analysis following the procedures as previously described [25]. Microbial samples (1 ml) were obtained following vigorous shaking of bottles to ensure representative sampling of both liquid and particle-associated fractions, and immediately snap-frozen in liquid nitrogen and stored at −80°C until subsequent RNA extraction. Additional 10 ml aliquots were collected and stored at −20°C for determination of nitrate and nitrite concentrations using the procedures as previously described [26]. The estimated net H_2_ production relative to the amount of total VFA produced (*R_NH2_*) was calculated according to the stoichiometric equation [27].

### Microbial RNA extraction, metatranscriptome sequencing, and mapping

Microbial RNA was extracted and quantified based on the protocol provided in Supplementary Materials and Methods. Metatranscriptome libraries were constructed using the stranded RNA library preparation procedure, using 5 μg of total RNA. Shortly, rRNA removal by Ribo-Zero rRNA Removal Kits from Illumina (San Diego, CA), fragmented using fragmentation buffer. Subsequent steps, including cDNA synthesis, end-repair, addition of A-bases, and ligation of the adapters, were conducted strictly according to the official protocol. Libraries were size-selected to obtain cDNA target fragments of 200–300 bp using 2% Low Range Ultra Agarose and amplified via 15 cycles of PCR using Phusion DNA polymerase (NEB). Metatranscriptome sequencing was performed by Shanghai Biozeron Biotechnology Co., Ltd (Shanghai, China), using the high-throughput sequencing (MGI-T7) platform, with PE150 mode according to the standard protocols.

The raw paired-end reads were trimmed and quality-controlled by Trimmomatic [28] (version 0.36) with parameters (SLIDINGWINDOW:4:15 MINLEN:75). High-quality reads were subsequently mapped to the host genome of sheep (*Ovis aries*, NCBI RefSeq GCF_016772045.1) using Bowtie2 [29] (version 2.3.5.1) to filter out host-derived sequences. A total of 947 590 414 cleaned reads were generated with an average of 78 965 868 ± 7 335 232 reads per sample for further analysis (Supplementary Table S2).

We first collected a total of 1382 metagenome-assembled genomes (MAGs) of putative methanogens and 21 methanogen genomes from publicly available archaeal genome catalogs derived from ruminant gastrointestinal tracts [30, 31]. These MAGs were dereplicated with dRep [32] (version 3.5.0) at 99% average nucleotide identity, yielding a curated, nonredundant set of 731 MAGs (completeness >50%, contamination <10%, Supplementary File S2). Clean reads were mapped to 731 MAGs using CoverM [33] (version 0.7.0), and transcripts per million (TPM) values were calculated to estimate the relative abundance of each MAG (Supplementary Fig. S6). Taxonomic classification of the MAGs was annotated by GTDB-Tk [34] (version 2.4.0) under default parameters, referencing the Genome Taxonomy Database [35] (GTDB, release 220). Open reading frames (ORFs) within MAGs were predicted by Prodigal [36] (version 2.6.3), then the resulting protein-coding sequences were annotated using KofamScan [37] (version 1.3.0) against the KEGG Orthology (KO) database through the exec_annotation pipeline [38] (Supplementary File S3). Multiple sequence alignments were refined using TrimAl [39] (version 1.4.rev15) to remove poorly aligned regions, after which phylogenetic trees were constructed using IQ-TREE2 [40] (version 2.4.0). We plotted the phylogenetic trees using iTOL [41] (version 6). Alpha diversity (Shannon index) and beta diversity metrics were calculated using the vegan package in R (https://www.r-project.org, version 4.4.1).

### Structural analysis through molecular docking with MCR

The MCR protein sequences (McrA, McrB, and McrG) were identified within the MAGs of putative methanogens through functional annotation by Prodigal [36] (version 2.6.3). These sequences were extracted and used as input for three-dimensional structure prediction via AlphaFold3 [42]. The quality of the predicted models was assessed based on AlphaFold3’s internal confidence metrics (Supplementary Fig. S7). To reconstruct the hexameric MCR complex with cofactors, each subunit structure was superimposed onto a crystallographic template (PDB ID: 1HBO) using a structural alignment protocol within the Maestro Small-Molecule Drug Discovery Suite (version 14.0). The MCR-predicted and 1HBO were aligned in Maestro (version 14.0) using the Protein Structure Alignment workflow with default settings, and root-mean-square deviation (RMSD) values were reported (Supplementary Table S3).

The small-molecule inhibitor 1,3-PDN was obtained from the PubChem Compound database (PubChem CID: 96257) in “.sdf” format. Ligand preparation involved conversion to a “.pdb” format, followed by energy minimization and protonation state adjustment using LigPrep. Simultaneously, the MCR structure predicted by AlphaFold3 was preprocessed using Protein Preparation Wizard, which included assigning bond orders, adding missing hydrogens, and optimizing side-chain conformations. Molecular docking was performed using Glide under standard precision mode [43]. The differences in docking-scores were computed using the Kruskal–Wallis rank-sum test. Binding poses were ranked by GlideScore, and only those showing a highly favorable orientation of 1,3-PDN (specifically positioning the nitrate ester moiety in close proximity to the nickel ion within the F_430_ cofactor) were retained for further visualization.

Potential functional pockets in the MCR structure were predicted using CASTpFold [44], which identifies and analyzes surface cavities and binding sites based on protein geometry. The resulting pocket models were subsequently visualized and examined in PyMOL [45] (version 2.6.0a0) for 3D inspection and confirmation of structural relevance. Amino acid sequences corresponding to the identified functional pockets were extracted and aligned to assess conservation across functional pockets of different MCR variants. Sequences corresponding to the identified functional pockets were aligned using the MUSCLE algorithm within MEGA [21] (version 11) under default parameters and visualized with Jalview [46] (version 2.11.4.0).

### Methanogenesis inhibition through culture of *M. olleyae*

The Experiment 3 was conducted to investigate the methanogenesis inhibition of 1,3-PDN through culture of *M. olleyae*. The *M. olleyae* was maintained in 10 ml Hungate tubes containing 5 ml of anaerobic medium, inoculated with 1 ml of actively growing culture [47]. Following 2 d of incubation at 39°C, the cultures were transferred into 150 ml serum bottles for treatment. Each bottle contained 25 ml of the culture and 25 ml of fresh medium under an H_2_/CO_2_ (80:20, v/v). In the treatment, 0.1 ml of 0.25 mM 1,3-PDN was added to obtain a final concentration of ~0.5 μM in the system after a 2-day incubation, whereas the control group received no additions (*n* = 5). Headspace gas (6 ml) was sampled every 2 d to determine CH_4_ concentration. The culture was harvested for proteomic analysis on Day 4.

Under cold conditions, proteomic samples were ground to powder and immediately transferred to centrifuge tubes (precooled with liquid nitrogen). Samples were lysed in SDT buffer containing 4% sodium dodecyl sulfate (SDS), 10 mM dithiothreitol (DTT), and 100 mM triethylammonium bicarbonate (TEAB), sonicated on ice (5 min), incubated (95°C, 8–15 min) and then placed on ice (2 min) [48]. The lysate was centrifuged (4°C, 12 000 × *g*, 15 min), and the supernatant was collected and treated with sufficient iodoacetamide (IAM) in the dark for 1 h [48]. Then samples were precipitated by adding 4 volumes of acetone (precooled), vortexing thoroughly, and incubating (−20°C, ≥2 h), followed by centrifugation (4°C, 12 000 *g*, 15 min) to collect the pellet [49, 50]. The pellet was washed with acetone (1 ml, precooled) and then fully dissolved in dissolution buffer (DB buffer) [49, 50]. Protein concentration was measured by Bradford protein quantitative kit. Twenty micrograms of each sample was loaded to 12% Sodium dodecyl sulfate polyacrylamide gel electrophoresis (SDS-PAGE) . After completing the electrophoresis, the samples were stained with Coomassie Brilliant Blue R-250 and decolorized until the bands became clearly visible. According to a previously described method [51], protein samples were digested with trypsin, and the eluents of each sample were collected and lyophilized. Samples were separated on a Vanquish™ Neo Ultra-High Performance Liquid Chromatography (UHPLC) system and subsequently analyzed on a Thermo Orbitrap Astral mass spectrometer equipped with an EASY-Spray electrospray ionization source operating in Data Independent Acquisition (DIA) mode. Raw data were acquired in .raw format.

We collected a total of 4459 protein sequences from the UniProt database (Supplementary File S4) (https://www.uniprot.org/). The raw files were analyzed using the DIA-NN search software. To improve the quality of the analytical results, the DIA-NN software further filtered the search results by retaining only credible Peptide Spectrum Matches with a confidence level of 99%. Only credible spectral peptides and proteins were retained, and False Discovery Rate (FDR) validation was performed to remove peptides and proteins with an FDR > 1%. Finally, 1658 proteins were matched across all samples, these proteins were subsequently queried against the KEGG database to obtain the corresponding gene names (Supplementary File S5).

### Nitrite inhibition assays based on nitrogen-equivalent 1,3-PDN concentration

The Experiment 4 was designed to investigate nitrite inhibition through culture of *M. olleyae*. Sodium nitrite was added at 1 μM, corresponding to the nitrogen-equivalent concentration of 1,3-PDN (0.5 μM, in Experiment 3), which contains two nitrate ester groups. Sodium nitrate (1 μM) was also included as a nitrogen-oxyanion control, given its minor effects on methanogens compared with other nitrogen-containing compounds [52, 53]. Specifically, 25 ml anaerobic tubes were prepared with 9.4 ml of fresh medium and inoculated with 0.5 ml of active *M. olleyae* culture. The tubes were incubated under an H_2_/CO_2_ atmosphere (80:20, v/v) at 39°C for 9 days with 0.1 ml 100 μM treatment solution added on day 3 (*n* = 3). Headspace gas was sampled every day for the measurement of CH_4_ concentration using a Shimadzu GC-14B gas chromatograph (Shimadzu, Kyoto, Japan), as described previously [54].

The Experiment 5 was designed to investigate nitrite inhibition through ruminal batch culture. Both sodium nitrite and sodium nitrate were added at a concentration of 24 μM (*n* = 6), corresponding to the nitrogen-equivalent concentration of 1,3-PDN (12 μM, in Experiment 2). The detailed procedures of ruminal batch culture were performed according to the Experiment 2.

### Data analyses

The kinetics of gH_2_ production (*V_H_*) were analyzed by NLREG [55] (version 5.4) using the equations reported previously [24] to obtain the fractional rate of gH_2_ formation (*k*) and utilization (*μ*), and discrete lag time (*lagH*). For intracellular accumulation data, a linear mixed model was employed to include treatment as a fixed effect. When sampling time was included, a linear mixed model was adopted with treatment and treatment by sampling time interaction as fixed effects, and sampling time as a repeated measurement. For metatranscriptomic data, a nonparametric Wilcoxon test was performed to assess differences between groups using JMP Pro (https://www.jmp.com/, SAS Institute, Cary, NC, USA, version 13.2.1). For proteomic data, differential expression analysis was conducted using the *limma* (Linear Models for Microarray Data) package in R. Multiple testing correction was applied using the FDR method. Differences with *P* < .05 were considered statistically significant.

## Results and discussion

### 1,3-PDN exhibits greater intracellular accumulation than 3-NOP

To compare the cellular permeability potential of 1,3-PDN and 3-NOP, we used a simplified archaeal membrane system [56] containing each inhibitor and simulated their transmembrane trajectories. Potential of mean force (PMF) profiles were then generated using umbrella sampling methods, in which the free-energy barriers represent the energetic cost required for membrane translocation [57, 58]. The PMF analysis showed that 1,3-PDN exhibited a markedly lower transmembrane energy barrier than 3-NOP (Supplementary Fig. S1B), suggesting more favorable transmembrane permeability, consistent with its increased hydrophobicity.

Given that the membrane model employed simplified the actual membrane composition and complexity of methanogens, we then conducted the intracellular accumulation assays for both inhibitors in *M. olleyae*, a rumen-derived hydrogenotrophic methanogen (Fig. 1A). The intracellular concentrations of 1,3-PDN and 3-NOP increased progressively over time, indicating that both inhibitors entered methanogen cells and accumulated intracellularly (Fig. 1B). Intracellular concentrations of 1,3-PDN were consistently higher than those of 3-NOP at all sampling time points (*P* < .001, Fig. 1B), and the apparent accumulation rate of 1,3-PDN was significantly faster than that of 3-NOP (*P* < .001, Fig. 2C). 1,3-PDN reached 4.6-fold higher intracellular accumulation per cell than 3-NOP across the three sampling time points (Fig. 2D). These results indicate that 1,3-PDN not only exhibits higher intracellular accumulation but also establishes intracellular enrichment more efficiently. Although intracellular accumulation does not directly quantify membrane permeability coefficients, the substantially higher cellular levels of 1,3-PDN under identical exposure conditions are consistent with enhanced cellular permeability relative to 3-NOP, aligning well with the computational simulations.

### 1,3-PDN inhibits CH_4_ production and reshapes H_2_ metabolism during ruminal fermentation

**Figure 3:**
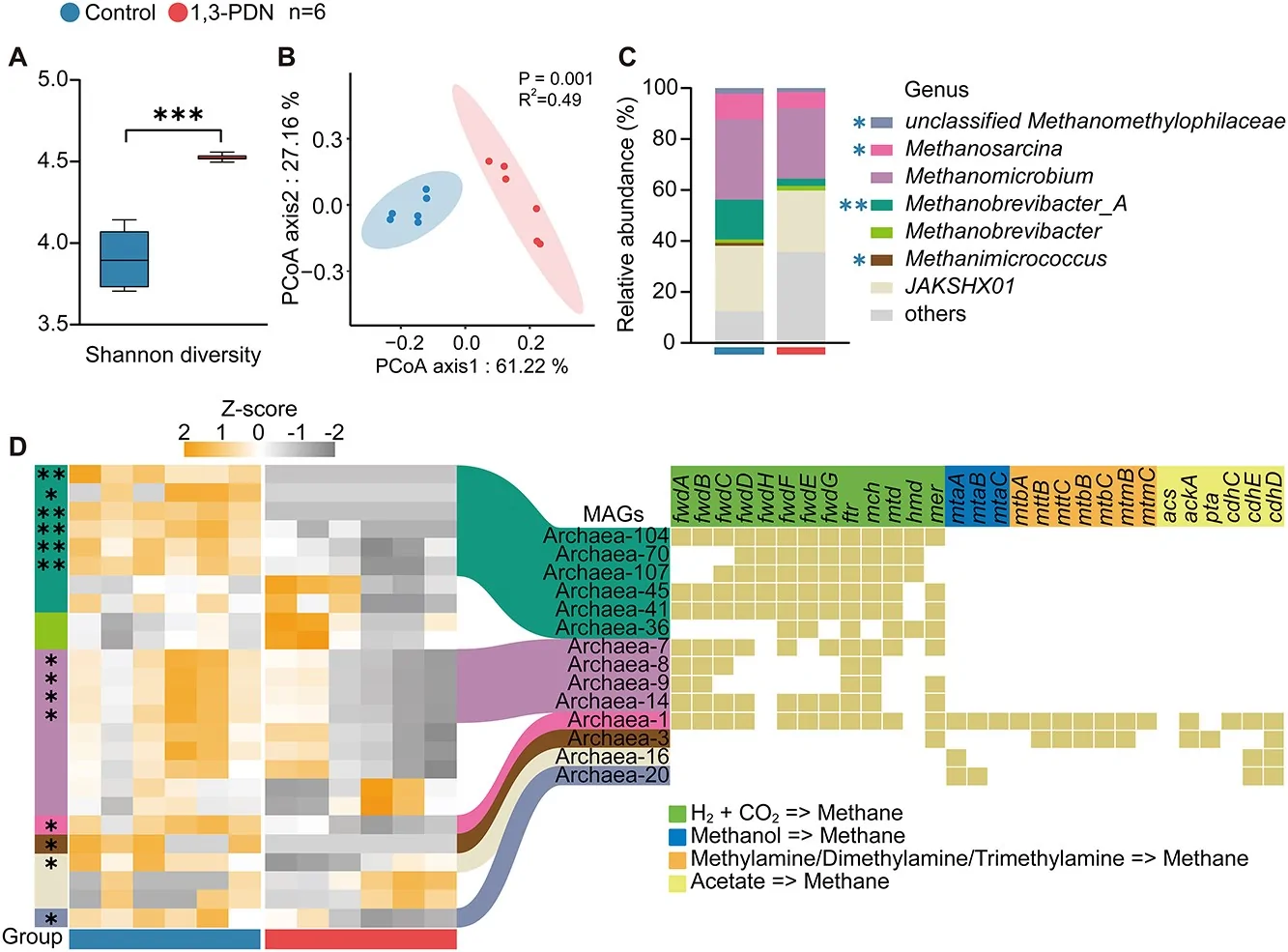
Effects of 1,3-PDN on the rumen methanogen community through metatranscriptomic sequencing. (A) Shannon index; (B) comparison of overall methanogenic community between supplementations by weighted UniFrac distances (PERMANOVA, *P* = .001, *R*^2^ = 0.49); (C) genus-level community of major archaea (relative abundance, %); (D) metatranscriptomic profiles (TPM, *Z*-score) of archaeal MAGs associated with methanogenesis, shown alongside the presence/absence matrix of key pathway genes: H_2_/CO_2_ to CH_4_ (*fmdABCDEFGH, ftr, mch, mtd, hmd, mer*), methanol to CH_4_ (*mtaABC*), methylamines/dimethylamines/trimethylamines to CH_4_ (*mtbA, mttBC, mtbBC mtmBC*), acetate to CH_4_ (*acs, ackA, pta, cdhCDE*). ^*^P < .05, ^**^P < .01, ^***^P < .001, *n* = 6; 1,3-PDN, 1,3-propanediol dinitrate.

To evaluate the inhibitory effect of 1,3-PDN on rumen methanogenesis, we conducted a ruminal batch culture fermentation experiment (Fig. 2A). Supplementation with 1,3-PDN significantly altered the kinetic of CH_4_ production, resulting in an ~55% reduction in cumulative CH_4_ yield after 48 h of ruminal incubation (Fig. 2B). The inhibitory effect was strongest during the early phase of fermentation but gradually declined over time and eventually disappeared (Fig. 3C). This temporal attenuation in efficacy may be attributed to the progressive transformation and depletion of 1,3-PDN in the fermentation system. This is consistent with previous observations for nitrate ester inhibitors, such as 3-NOP [59, 60].

Hydrogen (H_2_) is the primary intermediate metabolite generated during ruminal fermentation, and its rapid removal is essential to maintain low H_2_ partial pressures that support continuous microbial energy metabolism [61]. Hydrogenotrophic methanogens are the dominant methanogens in the rumen and efficiently consume H_2_ to reduce CO_2_ to produce CH_4_ [62]. Therefore, inhibition of methanogenesis leads to H_2_ accumulation in the ruminal fermentation system [63, 64]. Consistent with this mechanistic expectation, 1,3-PDN supplementation significantly increased cumulative H_2_ accumulation (i.e. 11-fold) after 48 h of ruminal batch culture (Fig. 3D). Such observed H_2_ accumulation could have great impact on microbial H_2_ formation and utilization. The fractional rate of H_2_ formation (*k*), which indicates the activity of H_2_-producing microbes [24], was significantly reduced following 1,3-PDN supplementation (Fig. 3E, Supplementary Table S4). This reduction in *k* likely reflects feedback inhibition of H_2_ formation and elevated H_2_ partial pressure [24], and corresponds with the observed increase in the lag time of gH_2_ accumulation (*lagH*) (Fig. 3E, Supplementary Table S4). Similarly, the fractional rate of H_2_ utilization (*μ*), reflecting the activity of H_2_-consuming microbes [24], was significantly decreased under 1,3-PDN supplementation (Fig. 3E, Supplementary Table S4). Taken together, these findings indicate that 1,3-PDN significantly inhibits hydrogenotrophic methanogenesis and alters H_2_ turnover during rumen fermentation.

Rumen H_2_ can influence the thermodynamics of carbohydrate fermentation pathways that produce VFAs [65]. Increased H_2_ partial pressure is positively associated with propionate and butyrate formation, and negatively associated with acetate formation [66]. Here, the 1,3-PDN group showed higher proportion of propionate, and a lower proportion of acetate and acetate-to-propionate ratio (*P* < .001, Supplementary Fig. S8A), which aligns well with patterns observed under other CH_4_ inhibitors, such as 3-NOP, seaweed-based additives and plant extracts (e.g. saponins and essential oils) [12, 67–70]. Carbohydrate fermentation to acetate releases net H_2_, whereas fermentation to propionate incorporates net H_2_ [25]. Increased H_2_ partial pressure can exert inhibit efficiency of H_2_ production, which was confirmed by decreased H_2_ production relative to the total VFA production (*R_NH2_*) under 1,3-PDN supplementation (*P* < .001, Supplementary Fig. S8B). Furthermore, H_2_ accumulation can also cause modifications in the alternative hydrogenotrophic H_2_ sinks [25, 71]. For example, acetogens belonging to uncultivated lineages of *Clostridia* become the most abundant hydrogenotrophs and stimulate reductive acetogenesis under the methanogenesis inhibition by 3-NOP [12]. Other hydrogenotrophs (e.g. sulfate reduction, nitrate ammonification, fumarate reduction) may respond weakly due to limitation of electron acceptors [12]. Overall, the H_2_ production through shift of carbohydrate fermentation pathway exceeds that of H_2_ consumption through hydrogenotrophic pathways, leading to decreased efficiency of H_2_ production and an 11-fold increase in H_2_ accumulation.

### 1,3-PDN alters archaeal community composition and suppresses key hydrogenotrophic taxa

To investigate how 1,3-PDN influences methanogenic community structure and activity, we performed metatranscriptomic sequencing of microbial samples. After removing host-derived reads, we obtained 71.07 Gb of high-quality paired-end sequences, averaging 5.92 Gb per sample (range: 5.13–6.79 Gb) (Supplementary Table S2). Clean reads were mapped to reference genomes of putative methanogens, including both MAGs and isolate genomes compiled from two recently published archaeal genome catalogs [30, 31]. From an initial set of 1403 dereplicated MAGs, 731 genomes of putative methanogens with medium to high quality were retained (completeness >50%, contamination <10%, Supplementary File S2). Among them, 127 MAGs showed detectable transcriptomic read mapping (Supplementary Fig. S9). Taxonomic annotation showed that these 127 MAGs were mainly affiliated with four methanogenic families: *Methanobacteriaceae* (*N* = 104), *Methanomethylophilaceae* (*N* = 9), *Methanomicrobiaceae* (*N* = 9), and *Methanosarcinaceae* (*N* = 5) (Supplementary Fig. S9).

1,3-PDN significantly altered the community of active methanogens, as indicated by increased Shannon index (*P* < .001, Fig. 3A). Principal coordinate analysis further revealed that 1,3-PDN reshaped the overall transcriptional profile of the methanogenic community (PERMANOVA: *P* = .001, *R*^2^ = 0.49; Fig. 3B). We performed significance testing on MAGs of putative methanogens with relative abundance exceeding 0.5%. At genus level, 1,3-PDN decreased relative abundances of *Methanobrevibacter_A* (−12.7%, *P* < .01), *Methanosarcina* (*P* < .05), *Methanimicrococcus* (*P* < .05), and unclassified *Methanomethylophilaceae* (*P* < .05) (Fig. 3C). At species level, 1,3-PDN decreased relative abundances of *Methanobrevibacter_A millerae_A, Methanobrevibacter_A sp900314615* (*P* < .01), *Methanobrevibacter_A sp902770715* (*P* < .01), *Methanosarcina barkeri* (*P* < .05), *Methanimicrococcus sp017613335* (*P* < .05), unclassified *Methanobrevibacter_A* (*P* < .01), and unclassified *Methanomethylophilaceae* (*P* < .05) (Supplementary Fig. S10). Ruminal hydrogenotrophic methanogens include the clade comprising sequences related to *M. smithii, M. gottschalkii, M. millerae*, and *M. thaurei* (SGMT), and the clade comprising sequences related to *M. ruminantium* and *M. olleyae* (RO) [72]. These two clades, collectively accounting for ~74% of archaeal community, play a critical role in ruminal methanogenesis [72, 73]. The relatively low abundance of methanogenic taxa (<6%, Supplementary File S6) within RO clade in our samples is most plausibly attributable to the sampling matrix, as the inoculum for ruminal fermentation assays was the filtered liquid phase, which excluded the solid phase with preferential enrichment of the RO clade [74]. The relative abundance of Archaea-104, annotated as *Methanobrevibacter_A millerae_A*, showed the greatest decrease among MAGs of putative methanogens following 1,3-PDN supplementation (−10%, *P* < .01; Fig. 3D, Supplementary File S6). *M. millerae*, which belongs to SGMT clade, has been reported to be an efficient CH_4_ producer, with its enrichment positively associated with high-CH_4_ emissions in the rumen [73, 75–77]. These findings indicate that 1,3-PDN exerts broad inhibitory effects on methanogenic archaea.

To assess the metabolic potential of the 14 MAGs of putative methanogens that were significantly inhibited, we examined the presence of key KEGG Orthology (KO) genes involved in the four canonical methanogenic pathways (Supplementary File S7), including the respective utilization of CO_2_, methanol, methylamine, and acetate [78]. Ten of the 14 MAGs (71%) encode genes associated with the hydrogenotrophic CO_2_-reducing methanogenic pathway, including genes encoding formylmethanofuran dehydrogenase (*fwdA, fwdB, fwdC, fwdD, fwdF, fwdH*), formyltransferase (*ftr*), methenyl-H_4_MPT cyclohydrolase (*mch*), methylenetetrahydromethanopterin dehydrogenase (*mtd*), 5,10-methenyltetrahydromethanopterin hydrogenase (*hmd*), and 5,10-methylenetetrahydromethanopterin reductase (*mer*) (Fig. 3D). Genes such as *fwd, mtd, hmd*, and *mer* are closely associated with H_2_ utilization [79, 80] (Supplementary Fig. S11). Therefore, when the hydrogenotrophic methanogenesis pathway is inhibited, H_2_ tends to accumulate in the system, consistent with the increased H_2_ accumulation observed in the rumen batch experiment (Fig. 2D). Four MAGs (i.e. Archaea-1, Archaea-3, Archaea-16, and Archaea-20) encoded key genes for the methanol- (i.e. *mtaA, mtaB*, and *mtaC*) and methylamine-utilizing pathways (i.e. *mtbA, mttB/C, mtbB/C*, and *mtmB/C*) (Fig. 3D). In addition, some MAGs (i.e. Archaea-1, Archaea-3, Archaea-16, and Archaea-20) also encoded genes associated with the acetoclastic methanogenic pathway, including acetyl-CoA synthetase (*acs* or *ackA/pta*) and acetyl-CoA decarbonylase/synthase complex genes (*cdhC, cdhD*, and *cdhE*) (Fig. 3D). Collectively, these results suggest that hydrogenotrophic methanogens, particularly *Methanobrevibacter_A*, represent the predominant active methanogens in the rumen and were the most strongly inhibited by 1,3-PDN.

### Molecular docking analysis reveals conserved binding of 1,3-PDN to the MCR catalytic site across diverse putative methanogenic genomes

As nitrate ester compounds have showed the function of MCR-targeted inhibition, we performed molecular docking analyses to explore the interaction between 1,3-PDN and the active site of MCR proteins, based on MCR structures predicted from the 14 representative MAGs of putative-methanogens. Ten high-confidence MCR structures were predicted using AlphaFold3, including two variants from Archaea-104 (Supplementary Fig. S7). Structural alignment with the crystallographic template 1HBO showed RMSD values <1 Å, supporting the structural reliability of the predicted models (Supplementary Table S3).

**Figure 4:**
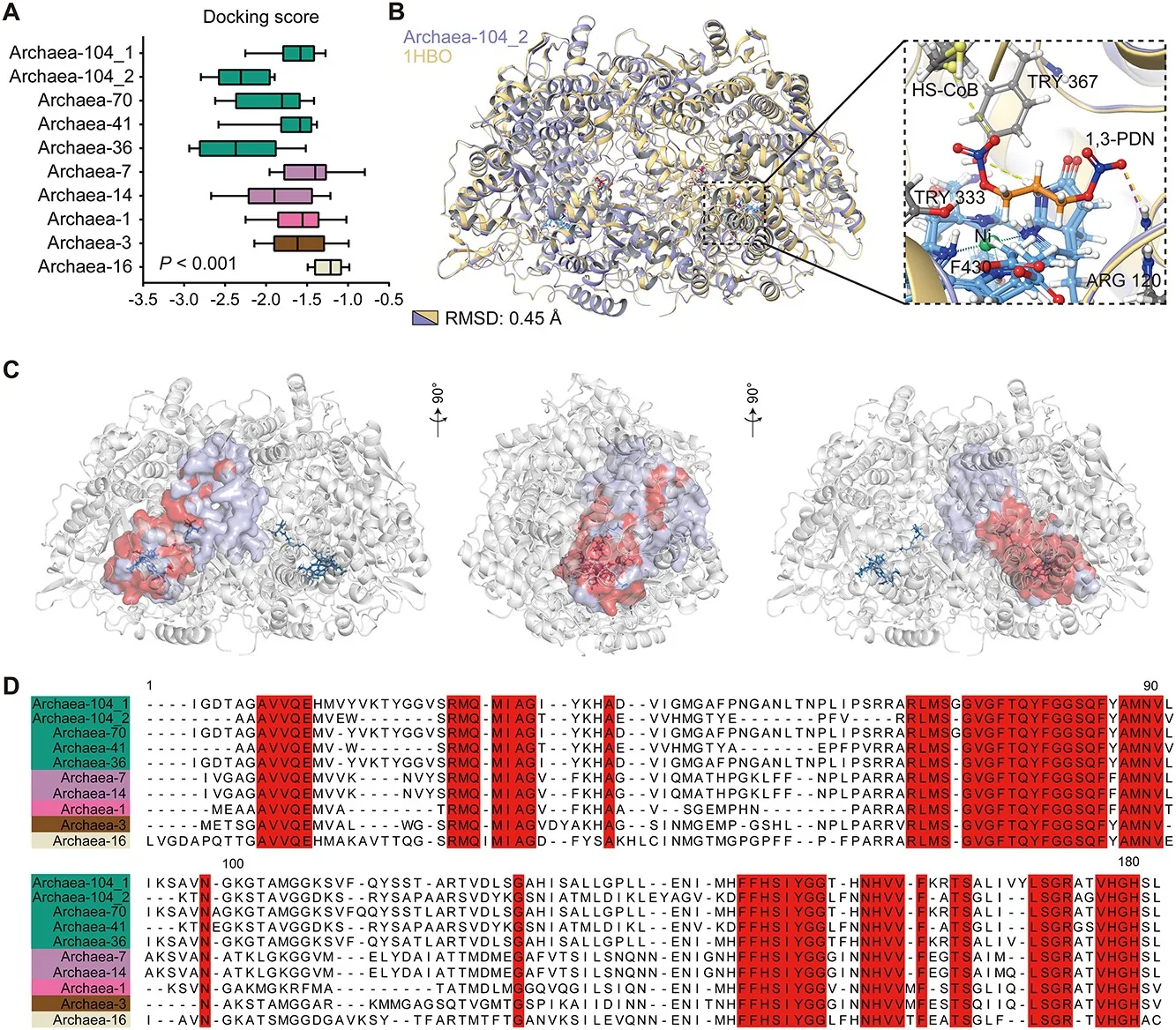
Structural basis of 1,3-PDN inhibition through molecular docking with predicted MCR complex in MAGs of putative methanogen. (A) Molecular docking scores for the binding of 1,3-PDN to the MCR complex with significant differences (*P* < .001); (B) structural alignment of MCR complex from Archaea-104_2 with reference crystal structure 1HBO. Close-up view reveals 1,3-PDN (orange sticks) binding near F_430_ cofactor (light-blue sticks) and key residues (Tyr333/Arg120/Tyr367, gray sticks), with hydrogen bonds (yellow dashes) and salt bridges (pink dashes). Atom coloring: N (dark blue), O (red), S (yellow), Ni(I) (green); (C) functional pocket of Archaea-104_2 and conserved MCR regions (red surface) and catalytic components (F_430_ and HS–CoB, dark-blue sticks); (D) sequence heatmap of functional-pocket amino-acid residues across MAGs of putative methanogen. Conserved regions are highlighted in red. RMSD, root-mean-square deviation; 1,3-PDN, 1,3-propanediol dinitrate.

Molecular docking revealed that 1,3-PDN binds at the MCR-active site with favorable docking scores. Among the tested models, Archaea-36 exhibited the strongest docking score, followed by Archaea-104_2, whereas Archaea-16 showed the weakest score (Fig. 4A). However, no significant correlation was found between docking scores and the experimentally measured methanogen inhibition degree (Supplementary Fig. S12), in aligned with uncorrelation between inhibition degree and scores of 3-NOP with the MCR catalytic sites [12]. These results suggest that factors beyond docking affinity, such as cellular uptake or metabolic context, may contribute to the differential inhibitory effects across methanogen species.

At the molecular level, 1,3-PDN was positioned directly above the F_430_ cofactor, occupying the canonical binding site of methyl-coenzyme M (CH_3_–S–CoM) (Fig. 4B). One of the nitrate nitrogen atoms in 1,3-PDN was located in close proximity to the nickel ion, resembling the binding mode reported for 3-NOP, which targets the same nickel ion [10]. Given their similar chemistry and binding mode in MCR catalytic center, 1,3-PDN may share aspects of the inhibitor mechanism proposed for 3-NOP, potentially involving redox interactions of the Ni center. In addition, the two termini of the 1,3-PDN molecule interacted with coenzyme B (HS–CoB), making contact with the conserved residue Arg120 (Fig. 4B), suggesting stabilization of the ligand–enzyme complex and enhanced inhibitory potency. These results indicate that 1,3-PDN can effectively bind to the MCR-active site and may competitively inhibit the natural substrate CH_3_–S–CoM.

To further investigate substrate access, we employed CASTpFold to characterize surface-accessible channels in the predicted MCR structures. The analysis identified an active-site cavity with an average volume of 2530.7 Å^3^ (range: 1728.7–2956.6 Å^3^), typically forming an “L”-shaped configuration. This cavity extends from the protein surface to the catalytic core, with one end opening to the exterior and the other enclosing the catalytic cofactors, including F_430_ and HS–CoB (Fig. 4C, Supplementary Fig. S13). Due to its hydrophobic properties, this pocket likely serves as a channel facilitating the diffusion of nonpolar small molecules from the surface into the enzyme’s active center [8, 81, 82]. Furthermore, despite some structural heterogeneities observed across MAGs of putative methanogens, several residues near the active site, including Arg120, Tyr367, and Tyr333, were found to be highly conserved (Fig. 4D). These conserved residues may provide a structural basis for efficient binding of 1,3-PDN to MCR in diverse putative methanogens. However, whether 1,3-PDN directly inhibits MCR activity remains to be determined using purified MCR and dedicated biochemical assays.

### 1,3-PDN inhibits hydrogenotrophic methanogenesis and alters methanogenesis-related proteomic profiles in *M. olleyae*

**Figure 5:**
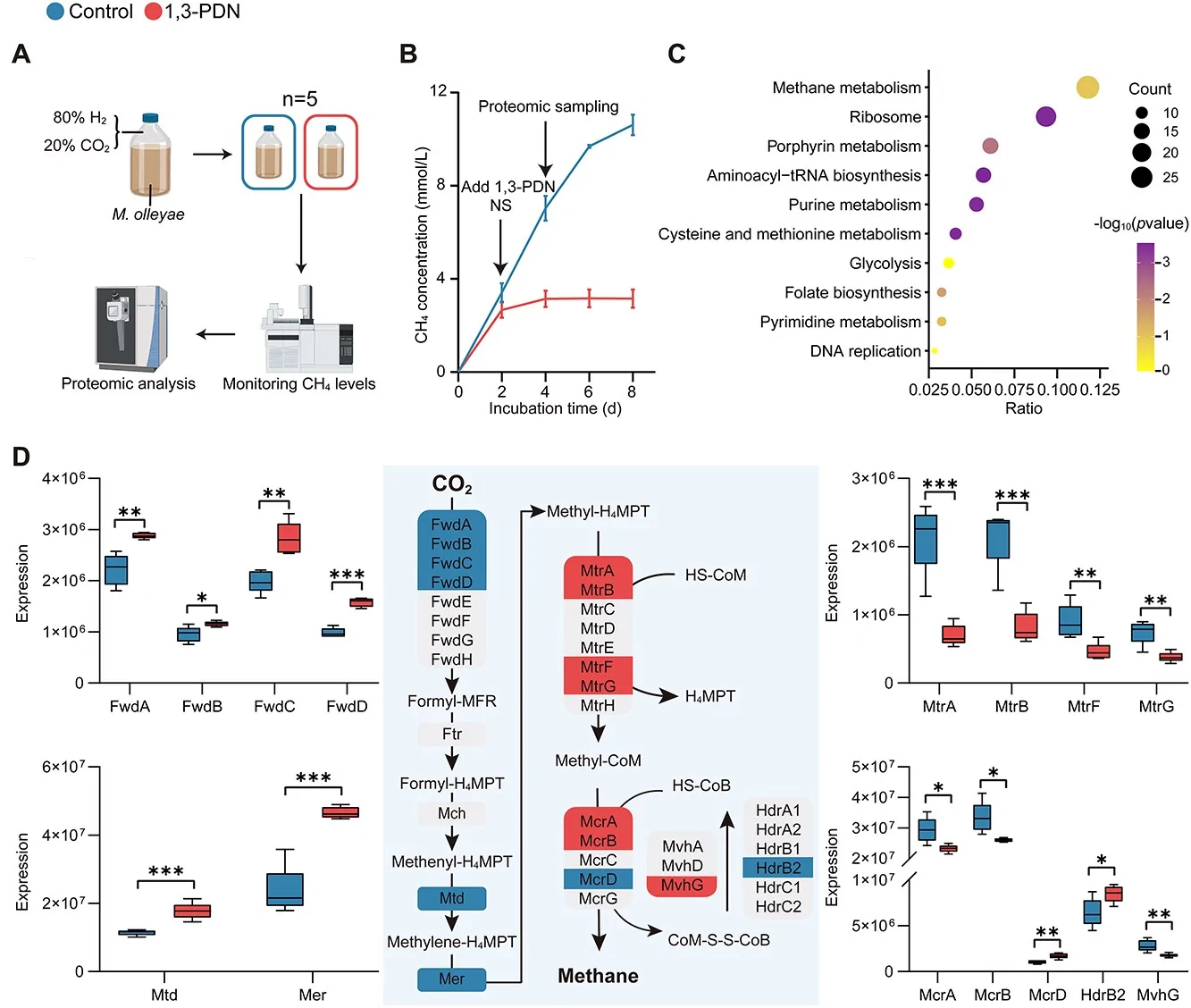
Effect of 1,3-PDN on methanogenesis in *M. olleyae* from the rumen of Hu sheep. (A) Workflow for the experimental design; (B) time–course curve of CH_4_ accumulation (mmol/L) during incubation (d); CH_4_ concentration was monitored throughout the incubation period. 1,3-PDN was added on Day 2 of incubation and samples collected on Day 4 were subjected to proteomic analysis. NS, No significant difference in CH_4_ concentration was observed among groups before inhibitor addition; (C) top 10 most affected KEGG pathways, including methane metabolism (ko00680), ribosome (ko03010), porphyrin metabolism (ko00860), aminoacyl-tRNA biosynthesis (ko00970), purine metabolism (ko00230), cysteine and methionine metabolism (ko00270), glycolysis (ko00010), folate biosynthesis (ko00790), pyrimidine metabolism (ko00240), and DNA replication (ko03030); (D) comparison of abundance for proteins involved in CO_2_/H_2_ methanogenesis pathway (KEGG pathway entry MD:M00567) between Control and 1,3-PDN supplementation. Data with error bars are expressed as mean ± standard error. Data with insignificant differences were present in Supplementary Fig. S14. ^*^P < .05, ^**^P < .01, ^***^P < .001, *n* = 5; 1,3-PDN, 1,3-propanediol dinitrate; *M. olleyae, Methanobrevibacter olleyae.* The schematic diagram was created using BioRender.

To further evaluate the inhibitory effect of 1,3-PDN on rumen-relevant hydrogenotrophic methanogenesis and explore its potential inhibitory mechanisms, we tested 1,3-PDN in *M. olleyae* and performed proteomic profiling of treated cultures (Fig. 5A). Following 1,3-PDN addition, CH_4_ production was significantly reduced. Although a slight increase in CH_4_ concentration was observed on Day 4 of incubation, no further CH_4_ accumulation was detected thereafter. By Day 8, supplementation with 0.5 μM 1,3-PDN resulted in a 70.3% reduction in CH_4_ production (*P* < .01, Fig. 5B), supporting the effective inhibition of hydrogenotrophic methanogenesis in this rumen-derived methanogen. However, potential community adaptation should be considered under prolonged exposure to methanogenesis inhibitors. For example, attenuation of CH_4_ mitigation has been reported during long-term 3-NOP supplementation in beef cattle, suggesting adaptative responses of the rumen methanogen community over time [83]. Therefore, long-term *in vivo* studies are needed to assess the durability, microbial responses, and practical applicability of 1,3-PDN-mediated CH_4_ mitigation.

The proteomic results indicated that 1,3-PDN downregulated 98 proteins (Supplementary File S8). Further KEGG pathway enrichment analysis indicated that the most affected pathways (top 10) included CH_4_ metabolism, ribosomal functions, porphyrin metabolism, and amino acid biosynthesis and metabolism (e.g. cysteine and methionine metabolism). The CH_4_ metabolism pathway involved the largest number of significantly changed proteins (Fig. 5C). Specifically, core catalytic subunits of the MCR complex, McrA and McrB, were significantly downregulated (*P* < .05), whereas McrD, a protein associated with MCR complex assembly [84], was significantly upregulated (*P* < .01) (Fig. 5D). This pattern likely represents a compensatory response to reduced MCR activity, with the cell attempting to restore complex assembly by increasing McrD expression. In parallel, upstream hydrogenotrophic enzymes including Fwd, Mtd, and Mer were significantly upregulated (Fig. 5D), consistent with known transcriptional responses to elevated H_2_ concentrations [79, 85, 86].

The tetrahydromethanopterin S-methyltransferase (MTR) complex also appears to be markedly affected by 1,3-PDN treatment. Expression of several key MTR subunits, including MtrA, MtrB, MtrF, and MtrG, were significantly downregulated following 1,3-PDN supplementation (*P* < .01) (Fig. 5D). These subunits form the central stalk of the trimeric and membrane-bound MTR complex, which plays a critical role in hydrogenotrophic methanogenesis [87]. MTR catalyzes the transfer of a methyl group from CH_3_–H_4_MPT to coenzyme M (HS–CoM), generating CH_3_–S–CoM, a direct substrate for the MCR complex [87]. Among the MTR subunits, MtrA binds a cobalamin-derived cofactor (cobamide), whose catalytic function depends on the reduced Co(I) state. The MTR activity requires this reduced state to facilitate its catalytic reaction. The presence of 1,3-PDN likely inhibited the catalytic process of MTR complex by oxidizing Co(I) to Co(II), rendering the enzyme catalytically inactive. This is consistent with the known high susceptibility to oxidizing agents of this enzyme, which stems from the low redox potential (*E*′ = −640 mV) of the cob(II)/cob(I) couple [88]. Accordingly, the 1,3-PDN may interfere with this process by oxidizing Co(I) to Co(II), thereby rendering the MTR complex catalytically inactive. This mechanistic inhibition is also supported by the altered expression of multiple proteins involved in cobalamin biosynthesis (Supplementary Fig. S15). Given that proteomic samples were collected 2 days after 1,3-PDN addition, compensatory response to prolonged inhibition can be observed in *M. olleyae*. For example, 1,3-PDN supplementation upregulated ribosomal proteins (Supplementary Fig. S16). Consistently, ribosomal proteins were broadly upregulated under prolonged methanogenesis inhibition in *M. barkeri* induced by high-ammonium stress, as previously reported [89]. These findings suggest that, under energy-limited conditions associated with CH_4_ inhibition, methanogens can enhance translation-related functions to maintain cellular functions and repair stress-induced damage [90]. Overall, these results suggest that the inhibitory effects of 1,3-PDN extend beyond the suppression of MCR expression, potentially involving broader disturbances to redox-sensitive methanogenic enzymes and cofactors, such as MTR, cofactor F_430_, and cobalamin.

### Nitrite contributes weakly to 1,3-PDN-mediated inhibition of ruminal methanogenesis

Nitrate ester compounds can undergo rapid bioconversion in rumen ecosystem, producing alcohol derivatives and nitrogen oxides, including nitrite [10]. Consistently, nitrite levels were significantly increased in 1,3-PDN-treated ruminal batch cultures (*P* < .05, Supplementary Fig. S8C), supporting the turnover of 1,3-PDN and release of nitrite during the fermentation. Nitrite can be absorbed across the rumen wall into the bloodstream and stimulates the conversion of hemoglobin into methemoglobin [91]. However, as a common rumen intermediate, elevated nitrite levels associated with 3-NOP supplementation shows to be safe and nontoxic to the animals [10, 11, 92]. Nevertheless, the safety associated with increased nitrite levels with the use of 1,3-PDN still warrants further evaluation, particularly under long-term feeding and *in vivo* conditions.

**Figure 6:**
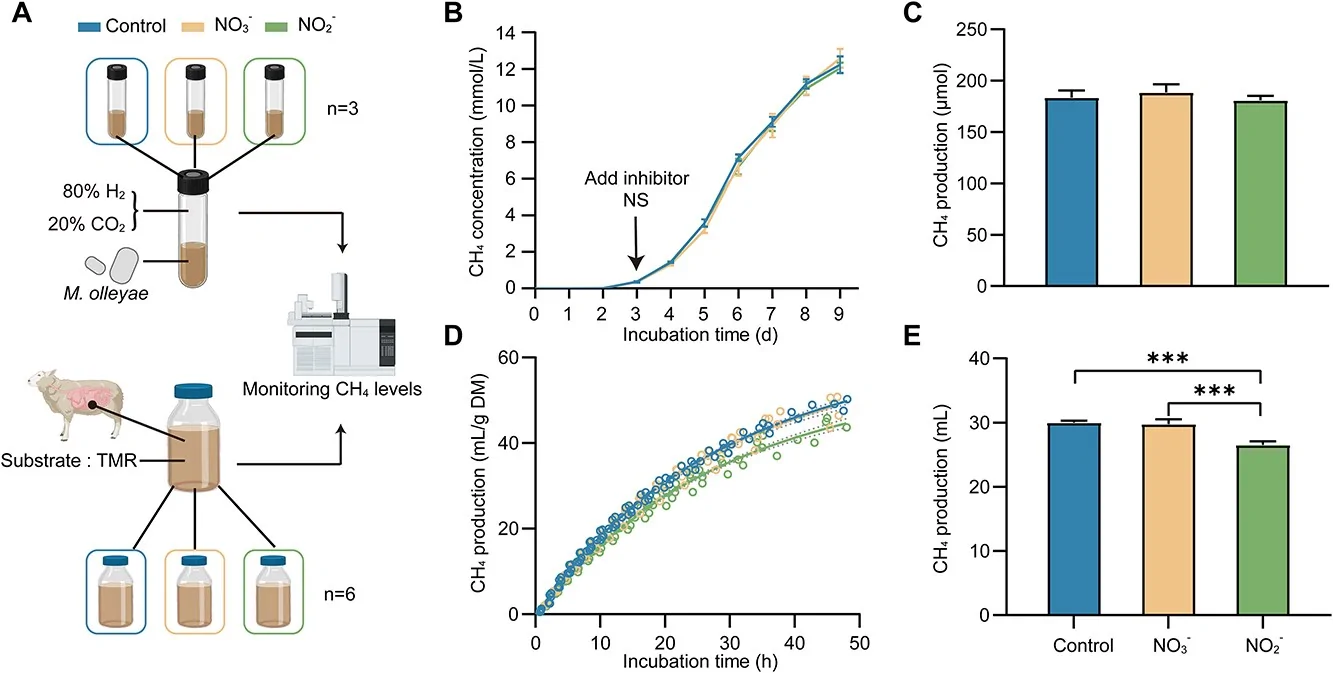
Effects of nitrite on methanogenesis in *M. olleyae* from the rumen of Hu sheep (B, C) and in ruminal batch culture (D, E). (A) Workflow for the experimental design. Control, nitrate (NO_3_^−^, negative treatment), and nitrite (NO_2_^−^). The CH_4_ concentrations were continuously monitored during the incubation. (B) Time-course curve of CH_4_ accumulation (mmol/L) during incubation (d, *n* = 3). Inhibitors were added on Day 3 of incubation. (C) Comparison of CH_4_ production (µmol) among treatment groups on Day 9. (D) Kinetics of CH_4_ accumulation (ml/g DM, *n* = 6). (E) Comparison of CH_4_ production (mL) among treatment groups at 48 h. The concentrations of nitrate and nitrite were determined based on a nitrogen-equivalent principle relative to the applied concentration of 1,3-PDN. NS, no significant difference in CH_4_ concentration was observed among groups before inhibitors addition. Data with error bars are expressed as mean ± standard error. ^***^P < .001; 1,3-PDN, 1,3-propanediol dinitrate; *M. olleyae, Methanobrevibacter olleyae*. The schematic diagram was created using BioRender.

To evaluate whether nitrite released from 1,3-PDN turnover could account for 1,3-PDN-mediated ruminal CH4 inhibition, we conducted nitrogen-equivalent nitrate and nitrite control experiments in the rumen-derived methanogen *M. olleyae* and in ruminal batch cultures (Fig. 6A). In *M. olleyae*, nitrite treatment hardly altered the kinetic of cumulative CH4 curves in comparison with that of the control and nitrate-treated group (Fig. 6B and C), whereas nitrogen-equivalent 1,3-PDN supplementation reduced CH_4_ production by 70.3% (Fig. 5B). Therefore, nitrite at the equivalent level expected from 1,3-PDN turnover did not reproduce the inhibitor effect of 1,3-PDN in this rumen-derived methanogen. Because nitrite-mediated inhibition of methanogenesis is dose-dependent and varies among methanogenic species [52, 53], the absence of significant inhibition by nitrite in this assay may be related to the relatively low concentrations and/or to the physiological characteristics of the strain used. In ruminal batch cultures, nitrite caused only a modest reduction in CH_4_ production with after 48 h of incubation (11.2%), whereas nitrogen-equivalent nitrate had little effect (Fig. 6D and E). This inhibition was substantially weaker than the 55% reduction observed with nitrogen-equivalent 1,3-PDN after 48 h of ruminal incubation (Fig. 2B). Studies report that nitrite-induced inhibition in mixed rumen systems is associated with H_2_ competition by nitrite-reducing bacteria, as well as its direct toxicity toward methanogenic archaea [91, 93]. Therefore, nitrite may contribute partially to 1,3-PDN-mediated CH_4_ inhibition in the rumen system, but it is unlikely to be the primary driver of the antimethanogenic effect of 1,3-PDN. However, we acknowledge that these experiments do not completely exclude the possibility for the contribution of other reactive nitrogen intermediates derived from 1,3-PDN degradation to methanogenesis inhibition, which needs further investigation.

## Conclusions

Replacing the –OH of 3-NOP with –ONO_2_ generates the more hydrophobic derivative 1,3-PDN, which exhibits greater intracellular accumulation in methanogenic cells. 1,3-PDN reduced cumulative CH_4_ production by ~55% after 48 h though ruminal batch culture, with the strongest inhibition occurring during early fermentation, while simultaneously causing an 11-fold increase in H_2_ accumulation. The reduced fractional rates of both H_2_ formation and utilization under increased H_2_ accumulation are consistent with suppression of hydrogenotrophic methanogenesis.

1,3-PDN significantly reshaped the active archaeal community through metatranscriptomic profiling, decreasing the abundance of major hydrogenotrophic taxa such as *M. millerae.* Molecular docking indicated that 1,3-PDN occupies the catalytic pocket of MCR and interacts with the amino acid residues Arg120, Tyr367, and Tyr333. Pure culture assays further confirmed strong inhibition of CH_4_ production in *M. olleyae*. Proteomic analyses revealed that this CH_4_ inhibition was associated with broader effects on redox-sensitive methanogenesis-related processes, including the downregulation of key subunits of MCR and MTR, as well as the effects on proteins involved in the biosynthesis of cofactors F_430_ and cobalamin. Nitrogen-equivalent assay indicated that nitrite can partially contribute to the methanogenesis inhibition and oxidative effects induced by 1,3-PDN.

Overall, our findings highlight that 1,3-PDN is a potent inhibitor of ruminal methanogenesis, with increased intracellular accumulation. These insights advance our mechanistic understanding of methanogenesis inhibition and thus provide a molecular foundation for the design of new inhibitors. However, the effectiveness of CH_4_ mitigation may vary over time and changes with alternation in dietary composition, more studies are needed to assess its safety, including the implications of the elevated nitrite concentrations from its metabolites, and the persistence of microbial community alterations induced by prolonged exposure. Additional investigation is also needed to determine whether other reactive nitrogen species potentially associated with 1,3-PDN contribute to its effects on methanogenic archaea. Differences in efficacy and persistence of 1,3-PDN compared with other inhibitors in the rumen microbial ecosystem, as well as the *in vivo* methane-inhibitory effect, still require further evaluation in the future.

## Supplementary Material

Supplementary_material_wrag200

## Data Availability

The data supporting this study are available within the article and the supplementary materials. Raw reads of metatranscriptomics sequencing have been deposited in the NCBI under the accession number PRJNA1347631. The mass spectrometry proteomics data are available to the ProteomeXchange Consortium (PXD070174).
