# Supplementary material for: Intracellular accumulation of hydrophobic 1,3-PDN inhibits methanogenesis in rumen methanogens
Source: ISME J. 2026 Jul 28;20(1):wrag200. doi: 10.1093/ismejo/wrag200 (PMC13525553; doi:10.1093/ismejo/wrag200)
Supplement: Supplementary file 1 [file supplementary_material_wrag200.zip › 20260723-1,3-PDN-Supporting Information (clean)_wrag200(1).docx]

**Supporting Information for**

**Intracellular accumulation of hydrophobic 1,3-PDN inhibits methanogenesis in rumen methanogens**

Bingzi Ouyang^1, 2#^, Yurong Cao^1, 2#^, Qiushuang Li ^1^, Shizhe Zhang^1, 2^, Xiang Zhou^1, 2^, Zhiwu Wu^1,2^, Xinyi Li^1,2^, Cancan Wang^1,2^, Rong Wang ^1,2^, Bo Fu ^3^, Xiaojia Liu^4^, Lixiang Yin^4^, Zhiliang Tan ^1, 2^, Kui Zhu^4^, Le Luo Guan ^5^, Xiumin Zhang^1, 2*^, Jie Li ^6*^, Min Wang ^1, 2*^

*^1^ State Key Laboratory of Forage Breeding-by-Design and Utilization, National Engineering Laboratory for Pollution Control and Waste Utilization in Livestock and Poultry Production, Institute of Subtropical Agriculture, Chinese Academy of Sciences, Changsha, Hunan 410125, China*

*^2^ University of Chinese Academy of Sciences, Beijing, 100049, China*

*^3^ Jiangsu Key Laboratory for Recognition and Remediation of Emerging Pollutants in Taihu Basin, School of Environmental Science and Engineering, Wuxi University, Wuxi, Jiangsu 214105, China*

*^4^State Key Laboratory of Veterinary Public Health and Safety, College of Veterinary Medicine, China Agricultural University, Beijing 100193, China*

*^5^ Faculty of Land and Food Systems, The University of British Columbia, Vancouver, BC, Canada V6T 1Z4*

*^6^ State Key Laboratory of Microbial Diversity and Innovative Utilization, Institute of Microbiology, Chinese Academy of Sciences, Beijing 100101, China*

^#^ These authors contributed equally to this work

* Corresponding author: Min Wang. Email: [mwang@isa.ac.cn](mailto:mwang@isa.ac.cn); Jie Li. Email: [lijie824@im.ac.cn](mailto:lijie824@im.ac.cn); Xiumin Zhang. Email: xmzhang@isa.ac.cn.

**This PDF file includes:**

Supplementary Material and Methods

Figures S1 to S17

Tables S1 to S7

Legends for Datasets S1 to S8

Supporting Information Text

Supplementary Material and Methods

Computation of the potential of mean force

To estimate the relative membrane translocation energetics of 1,3-PDN or 3-NOP, molecular dynamics (MD) simulations were performed with GROMACS [1] (version 2023.2). The archaeal membrane and small-molecule (AMSM) system was constructed by CHARMM-GUI Membrane Builder [2]. The membrane composition was constructed based on the model reported previously [3], with slight modification. The major components of the membrane are listed in Table S5. Potential of mean force (PMF) profiles along the membrane normal were calculated to approximate free-energy barriers associated with bilayer crossing. The archaeal membrane and small molecule (AMSM) system was first relaxed by energy minimization using the steepest descent algorithm. Long-range electrostatics were treated with Particle Mesh Ewald (PME) and real-space cutoffs of 1.2 nm, van der Waals interactions used a force-switch scheme between 1.0 and 1.2 nm. After minimization, the AMSM system was equilibrated for 4 ns at 312.15 K and 1 bar. Temperature control used the velocity-rescale thermostat (relaxation time 1.0 ps) and pressure was maintained by a C-rescale barostat under a semi-isotropic coupling scheme with a relaxation time of 5.0 ps and a compressibility of 4.5 × 10^-5^ bar^-1^ [4]. Standard neighbor-list settings with the Verlet scheme and a 1.2 nm list cutoff were applied. Positional restraints were kept during equilibration as in the input defines, and continuation was used to preserve velocities across stages. Bonds involving hydrogens were constrained with LINCS [5].

To generate starting configurations for umbrella sampling, a short pulling simulation was performed along the membrane normal (z-axis). The small molecule center-of-mass (COM) was coupled to an umbrella coordinate defined in the z-dimension with a harmonic potential (k = 1000 kJ·mol^-1^·nm^-2^). A constant pulling rate of 0.01 nm·ps^-1^ was applied to sweep the ligand through the bilayer center while keeping in-plane motion unconstrained (x-y free). Umbrella windows were then constructed along the reaction coordinate by fixing the COM separation at discrete z positions [6]. The windows were spaced at 0.05 nm intervals, with a total of 44 windows generated, covering the region from the initial position of the small molecule to the membrane center (Supplementary Figure S17). Each window was simulated for 5,000,000 steps (10 ns per window) with the same thermostat, barostat, PME electrostatics and nonbonded cutoffs as above. During umbrella sampling the pulling rate was set to zero, a static harmonic restraint about the target z was applied with the same force constant (k = 1000 kJ·mol^-1^·nm^-2^). The potential of mean force (PMF) along z was reconstructed with the weighted histogram analysis method (WHAM) as implemented in GROMACS [7]. Statistical errors were estimated using Bayesian bootstrap analysis (N = 100).

Isolation and purification of a hydrogenotrophic methanogen from rumen fluid of Hu sheep

Rumen fluid (2 mL) was obtained from a ruminal-cannulated Hu sheep and transferred into an anaerobic culture bottle containing previously autoclaved and deoxygenated medium (with resazurin as a redox indicator). The indicator appeared colorless under strictly anaerobic conditions, the medium composition is listed in Supplementary Table S6 [8] and was prepared following the procedure described in Supplementary Table S7. After inoculation, a gas mixture of H_2_/CO_2_ (80:20, v/v) was supplied at 0.1 MPa [9]. The culture was then incubated at 39 °C for 7 days as the first enrichment of methanogens. On day 7, the CH_4_ concentration in the headspace of each bottle was measured. The bottle with the highest CH_4_ level was identified, and 2 mL of the enriched culture from this bottle was aseptically transferred into fresh medium within an anaerobic chamber. After an additional 7 days of incubation, the procedure described above was repeated twice.

To further purify methanogens, a modified roll-tube technique based on the Hungate method was employed using an agar-amended anaerobic medium [10]. Following autoclaving, the medium was maintained at 50 °C in a water bath until use. Subsequently, 2 mL of enriched culture was aseptically inoculated into a roll-tube, thoroughly mixed, and rapidly solidified along the inner wall by swirling the tube in an ice-water bath. The headspace was flushed with an H_2_/CO_2_ (80:20, v/v) gas mixture, and the tubes were incubated at 39 °C for 10 days. Under anaerobic conditions and illumination with long-wave UV light, individual blue-green fluorescent colonies observed on the tube wall were selected based on fluorescence intensity and transferred into fresh liquid medium for further propagation [11].

An aliquot of the propagated culture with a hydrogenotrophic methanogen was subjected to another two rounds of roll-tube purification. In each round, ten colonies were randomly selected, re-cultured in liquid medium, and assessed for CH_4_ production. The culture exhibiting the highest CH_4_ concentration was used as the inoculum for the subsequent round of purification.

Microbial RNA extraction

Total RNA was extracted from microbial samples using E.Z.N.A® Stool RNA Kit, following the manufacturer's instructions. Genomic DNA contamination was subsequently eliminated by DNase I treatment (TaKaRa). RNA quality was assessed with an Agilent 2100 Bioanalyzer, and RNA concentration was quantified using the ND-2000 (NanoDrop Technologies). Only RNA samples meeting quality criteria (OD_260/280_ = 1.8-2.2, OD_260/230_ ≥ 2.0, RIN ≥ 6.5, and total amount >10 µg) were selected for metatranscriptomic library construction [12].

Figures

Figure S1. Generation of 1,3-PDN via replacing –OH of 3-NOP with –ONO_2_ substantially alters simulated permeation across the simplified archaeal membrane. A, Chemical formulas of small molecules (3-NOP: O_2_N–CH_2_–CH_2_–CH_2_–OH, 1,3-PDN: O_2_N–CH_2_–CH_2_–CH_2_–ONO_2_) and schematic of the functional-group substitution (i.e., –OH → –O–NO_2_) converting 3-NOP to 1,3-PDN; B, PMF of 3-NOP (kJ/mol) for the simulated permeants across archaeal membrane (yellow line), C, PMF of 1,3-PDN (kJ/mol) for the simulated permeants across archaeal membrane (red line). Statistical uncertainties are shown as shaded gray bands. The x-axis shows the center-of-mass distance of the permeants from the bilayer center along the membrane normal (nm). Free-energy barriers (ΔG, kJ mol⁻¹) for translocation are indicated by double-headed arrows. All PMF profiles were symmetrized about the bilayer center. PMF, potential of mean force; 3-NOP, 3-nitrooxypropanol; 1,3-PDN, 1,3-propanediol dinitrate.

**Figure S2. High-performance liquid chromatography of 1,3-PDN (100 μg/mL) monitored at 210 nm.**

Figure S3. Isolation and identification of a hydrogenotrophic methaogen. A, Schematic illustration of the workflow for the isolation of a hydrogenotrophic methanogen; B, Phylogenetic analysis based on 16S rRNA gene sequences of hydrogenotrophic methanogen isolates derived from the rumen of Hu sheep, using H_2_/CO_2_ as the sole energy and carbon source. Strain in bold is the isolate in this study. The family *Methanobacteriaceae* (dark green); the genus *Methanobrevibacter* (light green) and *Methanobacterium* (light blue); RO clade (pink).

**Figure S4. Identifying retention times of 3-NOP or 1,3-PDN, respectively, using gas chromatography-mass spectrometry.** A, Total ion chromatogram for the 3-NOP and 1,3-PDN; B, Full scan mass spectrum for 3-NOP; C, Full scan mass spectrum for 1,3-PDN. 3-NOP, 3-nitrooxypropanol; 1,3-PDN, 1,3-propanediol dinitrate.

**Figure S5. The temperature program for measuring 3-NOP or 1,3-PDN used gas chromatography-mass spectrometry.**

Figure S6. Workflow for MAGs of putative methanogens catalog curation and metatranscriptomic read mapping. Public MAGs of putative methanogens were downloaded from NCBI and dereplicated with dRep (v3.5.0; 99% ANI), yielded 731 nonredundant MAGs (CP > 50%, CT < 10%). In parallel, cDNA from 12 samples was sequenced to produce raw reads (78,965,868 ± 7,335,232 reads per sample), which were quality-controlled with Trimmomatic (v0.36), resulting in 947,590,414 clean reads. Clean reads were mapped to the curated methanogenic MAG catalog using CoverM (v0.6.1), resulting in 127 MAGs with mapped transcripts. (ANI, average nucleotide identity; CP, completeness; CT, contamination.)

Figure S7. AlphaFold3-predicted protein structures of MCR complex from 9 MAGs of putative methanogens inhibited by 1,3-PDN supplementation. The AlphaFold3-predicted structure of the MCR (Methyl-coenzyme M reductase) complex is shown on the left, with each subunit shown in a distinct color, while the corresponding predicted aligned error (PAE) plot is shown on the right, with both axes labeled by subunit identities. Color gradients in the PAE heatmap represent expected position errors, with darker green indicating higher confidence. The PAE plot was generated using the PAE Viewer web server. ipTM, interface predicted template modeling score; pTM, predicted template modeling score; 1,3-PDN, 1,3-propanediol dinitrate; MCR, Methyl-coenzyme M reductase.

**Figure S8. Effects of 1,3-PDN supplementation on fermentation end products in liquid phase.** A, Concentrations of total VFA (mmol/L), proportions of individual VFAs (mmol/L), and the acetate/propionate ratio; B, Net H_2_ production per 100 mol of VFA produced (*R_NH2_*); A, Concentrations of nitrate and nitrite (mg/L). Data with error bars are expressed as mean ± standard error. **P* < 0.05, ***P* < 0.01, ****P* < 0.001, n = 6; 1,3-PDN, 1,3-propanediol dinitrate.

Figure S9. Phylogenomic tree of the 127 MAGs of putative methanogens used in this study with the maximum-likelihood method (LG+F+I+R5, 1000 bootstrap), rooted at the midpoint. Tip colors denote families: Methanobacteriaceae (green), Methanomethylophilaceae (blue), Methanomicrobiaceae (purple), and Methanosarcinaceae (red).

**Figure S10. Effects of 1,3-PDN on archaeal community through metatranscriptomic sequencing at species level.** **P* < 0.05, ***P* < 0.01, n = 6; 1,3-PDN, 1,3-propanediol dinitrate.

Figure S11. Methanogenesis pathways from MAGs of putative methanogens that were inhibited by 1,3-PDN. The schematic shows four major CH_4_-producing pathways: utilization of H_2_/CO_2_ (green), methanol (blue), methylamine (orange), and acetate (yellow). Circled numerals indicate the presence of genes associated with the corresponding metabolic pathway in each MAG. Relevant genes along the pathways are highlighted in rose pink. Colored squares denote the genus-level taxonomy of each MAG. 1,3-PDN, 1,3-propanediol dinitrate.

Figure S12. Correlation between the degree of inhibition and docking scores of MAGs of putative methanogens that were inhibited by 1,3-PDN. The dashed lines indicate the 95% confidence interval of the linear regression model. 1,3-PDN, 1,3-propanediol dinitrate.

Figure S13. CASTpFold-predicted functional pocket of MCR complex from MAGs of putative methanogens inhibited by 1,3-PDN supplementation. The dark blue stick models represent F430 and HS-CoB; the light blue surface denotes the predicted functional pocket; the red surface highlights the conserved regions. MCR, Methyl-coenzyme M reductase; 1,3-PDN, 1,3-propanediol dinitrate.

Figure S14. Effect of 1,3-PDN on expression of proteins involved in CO_2_/H_2_ methanogenesis pathway. Data with error bars are expressed as mean ± standard error. **P* < 0.05, ***P* < 0.01, ****P* < 0.001, n = 5; 1,3-PDN, 1,3-propanediol dinitrate.

Figure S15. Effect of 1,3-PDN on expression of proteins involved in the porphyrin metabolism pathway. 1,3-PDN, 1,3-propanediol dinitrate. Data with error bars are expressed as mean ± standard error. **P* < 0.05, ***P* < 0.01, ****P* < 0.001, n = 5.

 **Figure S16. Effect of 1,3-PDN on expression of proteins involved in the ribosome pathway.** 1,3-PDN, 1,3-propanediol dinitrate. Data with error bars are expressed as mean ± standard error. **P* < 0.05, ***P* < 0.01, ****P* < 0.001, n = 5.

Figure S17. Umbrella-sampling histograms for membrane translocation by WHAM. A, 3-nitrooxypropanol; B, 1,3-propanediol dinitrate (1,3-PDN). Each colored curve is the count distribution for one umbrella window (N = 44 windows). WHAM, weighted histogram analysis method.

**Tables**

Table S1. Composition and ratios of the total mixed ration (TMR).

| **Item** | **Content** |
| --- | --- |
| Ingredient composition (g/kg DM) | 1000 |
| Forage content | 298.2 |
| Peanut vines | 149.1 |
| Leymus chinensis hay | 149.1 |
| Concentrate | 701.8 |
| Corn grain | 271.9 |
| Wheat bran | 49.7 |
| Corn gluten | 178.9 |
| Soybean phospholipid meal | 2.5 |
| Molasses | 6 |
| Soybean meal | 34.3 |
| Cottonseed meal | 34.3 |
| DDGS | 79.5 |
| Expanded urea | 5 |
| premix | 39.7 |

DDGS, Corn distillers dried grains with solubles.

Table S2 Summary of metatranscriptomic sequencing data.

| Item | Raw data | | | | | Clean data | | | | | |
| --- | --- | --- | --- | --- | --- | --- | --- | --- | --- | --- | --- |
|  | Total reads | Total bases | Q30 bases | Q30 rate | GC content | Total reads | Total bases | Q30 bases | Q30 rate | GC content | Proportion in raw data |
| Control1 | 69149372 | 10372405800 | 9838892810 | 94.86% | 45.70% | 69037342 | 10298741924 | 9770132709 | 94.87% | 45.67% | 99.84% |
| Control2 | 90672010 | 13600801500 | 12930466310 | 95.08% | 45.19% | 90562512 | 13477046330 | 12813583551 | 95.08% | 45.15% | 99.88% |
| Control3 | 80820506 | 12123075900 | 11535864506 | 95.16% | 44.38% | 80718132 | 12040747322 | 11458896999 | 95.17% | 44.34% | 99.87% |
| Control4 | 71110986 | 10666647900 | 10129534520 | 94.97% | 46.03% | 71034816 | 10610498338 | 10077436549 | 94.98% | 46.01% | 99.89% |
| Control5 | 68534008 | 10280101200 | 9730287972 | 94.65% | 45.56% | 68459794 | 10225756584 | 9680160412 | 94.67% | 45.54% | 99.89% |
| Control6 | 86277930 | 12941689500 | 12270020586 | 94.81% | 45.16% | 86189532 | 12834272624 | 12169082891 | 94.82% | 45.12% | 99.90% |
| 1,3-PDN1 | 79141138 | 11871170700 | 11287464327 | 95.09% | 46.04% | 79029872 | 11777352674 | 11199437185 | 95.09% | 46.00% | 99.86% |
| 1,3-PDN2 | 76898302 | 11534745300 | 10940369624 | 94.85% | 45.97% | 76791238 | 11415958352 | 10828529739 | 94.86% | 45.93% | 99.86% |
| 1,3-PDN3 | 84522540 | 12678381000 | 12057658906 | 95.11% | 47.46% | 84422002 | 12553753882 | 11939891669 | 95.11% | 47.43% | 99.88% |
| 1,3-PDN4 | 78478836 | 11771825400 | 11136230048 | 94.60% | 46.81% | 78399144 | 11697467788 | 11067067099 | 94.61% | 46.79% | 99.90% |
| 1,3-PDN5 | 88004010 | 13200601500 | 12569798138 | 95.22% | 46.86% | 87910324 | 13073128368 | 12449091601 | 95.23% | 46.82% | 99.89% |
| 1,3-PDN6 | 75109676 | 11266451400 | 10680205276 | 94.80% | 47.18% | 75035706 | 11176658774 | 10595704150 | 94.80% | 47.16% | 99.90% |

Table S3 The RMSD value between the MCR of MAGs of putative methanogens and the template protein 1HBO.

| MAG_ID | RMSD |
| --- | --- |
| Archaea-1 | 0.815 |
| Archaea-3 | 0.831 |
| Archaea-7 | 0.800 |
| Archaea-14 | 0.817 |
| Archaea-16 | 0.843 |
| Archaea-36 | 0.780 |
| Archaea-41 | 0.453 |
| Archaea-70 | 0.777 |
| Archaea-104_1 | 0.807 |
| Archaea-104_2 | 0.448 |

RMSD, root-mean-square deviation; MCR, Methyl-coenzyme M reductase

Table S4. Parameters for the kinetics of H_2_ production inhibited by 1,3-PDN through *in vitro* rumen batch culture.

| Item | Average | | SEM | *P*-Value |
| --- | --- | --- | --- | --- |
|  | Control | 1,3-PDN |  |  |
| *k* | 2.75 | 0.36 | 0.508 | 0.010 |
| *u* | 2.77 | 0.30 | 0.515 | 0.009 |
| *lagH* | 0.57 | 1.46 | 0.147 | <0.001 |

*k*, fractional rate of H_2_ formation (h^−1^); *μ*, fractional rate of H_2_ re-solution (h^−1^); *lagH*, the lag time of H_2_ accumulation (h); 1,3-PDN, 1,3-propanediol dinitrate

Table S5. Composition and quantity of the archaeal membrane.

| **Lipid name** | **Lipid head/tail** | **Quantity** |
| --- | --- | --- |
| PGAR | PG-AR | 47 |
| PGMAR | PGP-MeAR | 17 |
| ARCL2 | BPG (C25, C20, C25, C20) | 7 |
| AROL | Core-AR (Archaeol) | 8 |

Table S6. Hydrogenotrophic methanogens medium.

| **Medium component** | **Amount** |
| --- | --- |
| Distilled water | 800 mL |
| Clarified rumen fluid | 140 mL |
| KH_2_PO_4_ | 1.4 g |
| (NH_4_)_2_SO_4_ | 0.6 g |
| KCl | 1.5 g |
| NaHCO_3_ | 4.2 g |
| L-cysteine·HCl·H_2_O | 0.5 g |
| Yeast extract | 2.5 g |
| 0.4 mol/L Mg/Ca mix (magnesium/calcium solution) | 20 mL |
| Mixed solution of 3 mol/L sodium formate, 1 mol/L sodium acetate, and 1 mol/L methanol | 40 mL |
| Sodium sulfide–cysteine reducing agent | 5 mL |
| Trace element solution | 1 mL |
| 0.1% (w/v) resazurin solution | 2 mL |
| **Additives Before Use** | **Dosage** |
| Vitamin 10 mix solution (optional) | 0.1 mL per 9.5 mL of culture medium |
| 1,000,000 IU/mL penicillin + 2,000,000 IU/mL streptomycin (freshly prepared, use immediately) | 0.1 mL per 9.5 mL of culture medium |

Table S7. Medium preparation procedures.

| **Procedures** | **Description** |
| --- | --- |
| Dissolution | Add all the components above into a 2.5 L nitrile anaerobic bottle and stir with a magnetic stirrer until fully dissolved. |
| Filtration | Filter the solution after complete dissolution. |
| CO_2_ sparging and heating | Place the filtered solution in a water bath at 80–90°C, continuously sparging with CO₂. Observe the medium color change from blue to pink, and finally to golden yellow. |
| Dispensing | Using a 20 mL syringe, dispense the medium into anaerobic tubes containing 0.15 g agar powder (1.5%), 10 mL per tube. |
| Sealing | Cap the tubes with butyl rubber stoppers and plastic caps. |
| Vacuum and sterilization | Apply a vacuum using a vacuum pump, then autoclave. Store in the dark. |

**Dataset S1 (separate file).** 16S rRNA amplicon sequence from the hydrogenotropic methanogen isolate and blast results.

**Dataset S2 (separate file).** Taxonomy, quality, and source of MAGs of putative methanogens used in this study.

**Dataset S3 (separate file).** Key KEGG Orthology (KO) genes involved in the four major CH_4_-producing pathways—the utilization of CO_2_, methanol, methylamine, and acetate.

**Dataset S4 (separate file).** The protein sequences retrieved from the UniProt database.

**Dataset S5 (separate file).** Protein sequences identified in the samples and their corresponding gene names annotated in KEGG.

**Dataset S6 (separate file).** Abundance (TPM) of MAGs of putative methanogens in metatranscriptomes.

**Dataset S7 (separate file).** KO distributions for 127 MAGs of putative methanogens linked to KEGG pathway ko00680.

**Dataset S8 (separate file).** Expression of proteins in the proteome.

**References**

1.	Pronk S, Páll S, Schulz R *et al.* Gromacs 4.5: A high-throughput and highly parallel open source molecular simulation toolkit. *Bioinformatics*. 2013;**29**:845-54 <https://doi.org/10.1093/bioinformatics/btt055>

2.	Jo S, Kim T, Iyer VG *et al.* Charmm-gui: A web-based graphical user interface for charmm. *J Comput Chem*. 2008;**29**:1859-65 <https://doi.org/10.1002/jcc.20945>

3.	Pogozheva ID, Armstrong GA, Kong L *et al.* Comparative molecular dynamics simulation studies of realistic eukaryotic, prokaryotic, and archaeal membranes. *J Chem Inf Model*. 2022;**62**:1036-51 <https://doi.org/10.1021/acs.jcim.1c01514>

4.	Bussi G, Donadio D, Parrinello M. Canonical sampling through velocity rescaling. *J Chem Phys*. 2007;**126**:014101 <https://doi.org/10.1063/1.2408420>

5.	Hess B, Bekker H, Berendsen HJC *et al.* Lincs: A linear constraint solver for molecular simulations. *Journal of Computational Chemistry*. 1997;**18**:1463-72 <https://doi.org/https://doi.org/10.1002/(SICI)1096-987X(199709)18:12><1463::AID-JCC4>3.0.CO;2-H

6.	Lemkul JA. From proteins to perturbed hamiltonians: A suite of tutorials for the gromacs-2018 molecular simulation package [article v1.0]. *Living Journal of Computational Molecular Science*. 2019

7.	Hub JS, de Groot BL, van der Spoel D. G_wham—a free weighted histogram analysis implementation including robust error and autocorrelation estimates. *Journal of Chemical Theory and Computation*. 2010;**6**:3713-20 <https://doi.org/10.1021/ct100494z>

8.	Miller TL, Wolin MJ. A serum bottle modification of the hungate technique for cultivating obligate anaerobes. *Appl Microbiol*. 1974;**27**:985-7 <https://doi.org/10.1128/am.27.5.985-987.1974>

9.	Balch WE, Fox GE, Magrum LJ *et al.* Methanogens: Reevaluation of a unique biological group. *Microbiol Rev*. 1979;**43**:260-96 <https://doi.org/10.1128/mr.43.2.260-296.1979>

10.	Hungate RE. The anaerobic mesophilic cellulolytic bacteria. *Bacteriol Rev*. 1950;**14**:1-49 <https://doi.org/10.1128/br.14.1.1-49.1950>

11.	Edwards T, McBride BC. New method for the isolation and identification of methanogenic bacteria. *Appl Microbiol*. 1975;**29**:540-5 <https://doi.org/10.1128/am.29.4.540-545.1975>

12.	Zhang C, Shi J, Wang S *et al.* Transcriptome for small yellow follicles reveals bone morphogenetic protein 15 involved in chicken follicle selection. *Animal Advances*. 2025;**2**:e016 <https://doi.org/10.48130/animadv-0025-0010>
